# Tracking Cell Movement in Two‐Dimensional, Fragmented Microcosms Reveals Dispersal Syndromes and Strategies in *Tetrahymena thermophila*

**DOI:** 10.1002/ece3.73092

**Published:** 2026-02-12

**Authors:** Florent Manzi, Victor Brans, Michaëlla Dacek, Staffan Jacob, Nicolas Schtickzelle

**Affiliations:** ^1^ Earth and Life Institute, Biodiversity Research Centre Université Catholique de Louvain Louvain‐la‐Neuve Belgium; ^2^ Centre National de la Recherche Scientifique (CNRS) Station d'Écologie Théorique et Expérimentale (UAR2029) Moulis France

**Keywords:** ciliates, dispersal syndromes, emigration, habitat fragmentation, immigration

## Abstract

A major challenge in dispersal ecology consists of testing whether distinct sets of phenotypic traits are associated with the three main phases of dispersal, requiring direct observations of disperser movements during emigration, transience, and immigration. Although freshwater ciliates have been used as a model in artificial dispersal landscapes for over 15 years, most studies would relate dispersal propensity to phenotypic traits measured at the end of dispersal assays. Using ‘two‐dimensional’ fragmented microcosms, abundance, movement and morphology data of *Tetrahymena thermophila* were collected at numerous time points throughout 6.5 h‐long dispersal assays. Data were compared across distinct zones (‘Start’ and ‘Target’ patches, connected by a ‘Corridor’) to identify shifts in the mean value and distribution of dispersal‐related traits. Inference on the existence of dispersal decisions was obtained by comparing these results to similar outputs generated by a ‘null’ movement model (without decision rules). Five genotypes were used, among which two strategies were identified: swimming speed and linearity either increased (‘hump’) or decreased (‘slope’) during transience, while both traits generally decreased at immigration. Doubling the length of corridors (10 mm vs. 20 mm) modified dispersal timing, but did not affect emigration rates. Simulated data predicted a shift towards increased velocity at immigration; however, the opposite was found in most strains, suggesting a plastic inducement of typical foraging movements after settling in the ‘Target’ patch. Since a ‘snapshot’ approach was used (capturing sparse movement sequences throughout the dispersal process instead of prolonged tracking), phenotypic plasticity could not be confirmed with certainty; however, the hypothesis of strict spatial sorting was insufficient to explain movement patterns. Overall, our results hint at the plastic and reversible nature of dispersal syndromes displayed by *T. thermophila* across fragmented landscapes, which bears significance in the context of habitat loss and the maintenance of metapopulation stability.

## Graphical abstract

Dispersal strategies of the ciliate *Tetrahymena thermophila* were investigated using ‘two‐dimensional’ artificial landscapes, allowing for camera tracking of individual cell trajectories during all steps of the dispersal process. Strains showed distinct strategies at emigration, either increasing or decreasing movement traits (swimming speed and linearity) in the corridor; all genotypes decreased those traits at immigration. Doubling the length of corridor modified the timing of dispersal, but did not affect emigration rates.

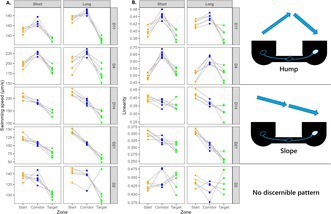

## 1 Introduction

Anthropogenic disturbances such as agricultural land use, resource extraction and urbanization are causing a loss of habitat at the global scale, entailing both a net reduction in the total surface of suitable habitats and their fragmentation per se, with increasing distance between patches (Laurance 2014; Newbold et al. 2015; Prakash and Verma 2022). The latter is expected to amplify the costs of movement, which should result in decreased dispersal rates of natural populations (Schtickzelle and Baguette 2003; Fahrig 2007; Haddad et al. 2015; Cote et al. 2017). Still, long‐distance movements of individuals (and thus gene flow) through space remain crucial for species to subsist as stable metapopulations across fragmented environments and maintain genetic diversity, consequently alleviating risks of genetic drift and species extinction (Trakhtenbrot et al. 2005; Lawton et al. 2011). Not all conspecifics that make up a population are capable nor necessarily willing to undertake dispersal movements, however, as both intra‐specific and intra‐population variability in dispersal propensity exist, on which natural selection can act upon (Harrison 1980; Kisdi 2016). In the wake of a dispersal event, individuals can be categorized as either ‘dispersers’ or ‘residents’, depending on whether they have dispersed. The question as to whether that distinction somehow pre‐exists (and can be detected) in populations, as well as which sets of traits distinguish individuals of both types remains a topical subject in the study of dispersal ecology (Cote et al. 2017; Jacob et al. 2020; Raffard et al. 2023).

A promising biological model to study resident‐disperser differences are freshwater ciliates: capable of directional swimming motivated by chemotaxis and various environmental cues, the North American species *Tetrahymena thermophila* inhabits sparse ‘hotspots’ within ponds and lakes, mostly patches of submerged vegetation that provide cover and a high concentration of bacterial food (Doerder and Brunk 2012). Since they rely on distant hydrophytes as suitable patches of habitat, the water column in turn acts as a less hospitable ‘matrix’, which ciliates can move through. Therefore, environmental shifts such as warming or eutrophication are likely to affect the fragmentation level experienced by ciliates in nature, by altering the community composition and abundance of sheltering plant species, including a loss of submerged macrophytes in favor of emergent species and phytoplankton (Ansari et al. 2010; Xing et al. 2013; Lind et al. 2022). Because monitoring dispersal movements of ciliates in nature remains beyond the scope of current research, artificial microcosms have served as a useful alternative to study those problematics and simulate global change in controlled settings (Altermatt et al. 2015). Conveniently, those allow full control over the genetic background of studied individuals, environmental conditions that can be manipulated and stabilized over prolonged periods of time, and also facilitate measurements of relevant response traits.

During the past decade, significant progress has been made in documenting resident‐disperser differences in common laboratory ciliates. This has enabled the detection of several dispersal syndromes (i.e., positive associations of certain phenotypic traits with dispersal behavior; Ronce and Clobert 2012), specifically in the *Tetrahymena* genus. For instance, proficient dispersers tend to display a more elongated shape, smaller size and greater activity rate than resident conspecifics (Nelsen and Debault 1978; Pennekamp et al. 2014), although this pattern can differ across strains (Jacob et al. 2016; Jacob et al. 2020). The current body of knowledge on this topic has been acquired through a relatively consistent set of methods: *Tetrahymena* spp. and other laboratory ciliates (e.g., *Paramecium caudatum* ) were placed in artificial landscapes consisting of two or more habitat patches (plastic microtubes) connected by a horizontal silicon tube serving as a corridor. Access to the corridor was authorized for a given span of time (typically a few hours in those genera); at the end of this allocated period, the respective abundance, behavior and aspect of cells found in the ‘Start’ and ‘Target’ patches could be estimated (e.g., Fjerdingstad et al. 2007; Fellous et al. 2011; Pennekamp et al. 2014; Laurent et al. 2020). Distinct iterations of this method have proven efficient in prior experimental studies, allowing to shed light on complex processes such as context‐dependent dispersal, habitat choice based on thermal preference, as well as social aggregation and cooperation in this model (e.g., Chaine et al. 2010; Pennekamp et al. 2014; Jacob et al. 2016; Jacob et al. 2018; Laurent et al. 2020). Nevertheless, a major limitation of the aforementioned devices resides in their three‐dimensional nature, which prevents camera tracking of individual cells during dispersal (but see Cheong et al. 2015); another lies in the difficulty to reconstruct trajectories for a large number of cells across dispersal distances that exceed a few millimeters. Consequently, the exact movement patterns and morphology of cells crossing the corridor, as well as the temporality of dispersal events have thus far constituted a ‘black box’.

Though it is sometimes modelized as a simple binary decision (i.e., ‘leave’ or ‘stay’), dispersal is more aptly described as a continuous process comprising three steps: emigration, transience in the matrix, and immigration (Ims and Yoccoz 1997; Bowler and Benton 2005). Each of these stages requires distinct sets of dispersal decisions, informed by a combination of internal‐state variables and environmental cues, taken at discrete points in time and at the level of the individual (Clobert et al. 2009; Mabry and Stamps 2008). Owing to the aforementioned technical limitation, most experimental studies on ciliates would relate dispersal propensity to associated phenotypic traits after the entirety of the dispersal process had already taken place, based on the final position of ‘resident’ and ‘disperser’ cells. To better understand underlying processes that lead to population‐wide dispersal events, it is necessary to contrast phenotypic traits associated with dispersal (morphology and swimming behavior) in cells detected before emigration, during transience, and after immigration. Such a method is also required to disentangle the ‘egg‐and‐hen’ dilemma surrounding dispersal syndromes, as it is still unclear whether reported syndromes arise from a sorting of proficient phenotypes that pre‐exist in the starting population, or rather from plastic changes in cell phenotypic traits that could occur before, during or after dispersal (see for instance Jacob et al. 2020; Cayuela et al. 2022).

Here, a highly automated setup for imaging and analysis of ciliate dispersal movements was used in tandem with a novel category of low depth dispersal microcosms, hence referred as ‘two‐dimensional’. These devices allow cells to disperse across a fragmented landscape (i.e., two patches connected by a corridor) while gathering abundance, morphology and movement data at numerous time points throughout prolonged dispersal assays. Five strains of *T. thermophila* were tested within bidimensional landscapes that differed in the length of their corridor (either 10 mm or 20 mm). This factor was manipulated to investigate how distinct fragmentation levels can affect dispersal syndromes and decisions in those novel devices, a parameter that had been the topic of prior experimental studies. Jacob et al. (2020) previously found that fragmentation can modulate dispersal syndromes pertaining to morphology and movement traits in *T. thermophila* , with an increase in matrix harshness seemingly accentuating the differences in cell elongation and velocity observed between residents and dispersers of the same lineage. Experimenting with a distinct aspect of fragmentation (inter‐patch distance), Laurent et al. (2020) found that a two‐fold increase in corridor length differentially affected emigration and immigration decisions taken by *T. thermophila* . Building upon such studies, a similar landscape configuration was implemented in two‐dimensional microcosms to investigate (i) whether distinct dispersal syndromes could be detected at each main phase of dispersal and (ii) whether those were maintained across varying degrees of landscape fragmentation. Finally, inference on the existence of dispersal decisions was obtained by comparing emigration, movement and spatial localization data to similar outputs generated under a ‘null’ simulation model (without decision rules).

## 2 Methods

### 2.1 Model Species, Strains, and Culture Conditions

*Tetrahymena thermophila* (Ciliophora: Hymenostomatidae) is a 20–50 μm long unicellular ciliate inhabiting freshwater ponds and streams of North America. Laboratory stock cultures were kept in sterile 24‐well plates (Greiner Bio‐One CELLSTAR) filled with 2 mL of axenic PPYE growth medium (0.6% Difco Proteose Peptone and 0.06% Difco Yeast Extract) and maintained inside a Sanyo MIR‐554 incubator with a 12:12 light–dark cycle (Brans et al. 2024). Cultures were kept at a constant 23°C temperature to match room temperature of the laboratory experienced by cells during dispersal assays. Five clonal lineages were used (D2, D4, D11, D14 and D21). These were chosen in a semi‐random manner from a wider collection of strains cryoconserved in our laboratory, with the intent to cover a wide range of traits such as swimming speed and dispersal rates, as determined from prior studies in three‐dimensional microcosms (for a detailed characterization of each strain, see Table S1 in Appendix S1). Cryoconserved samples were thawed and six independent replicates (wells) were created for each strain. Those 30 cultures were renewed each week by transferring 20 μL from the previous well into 2 mL of fresh, sterile PPYE medium.

### 2.2 Assembly and Characteristics of the Dispersal Plates

**FIGURE 1:**
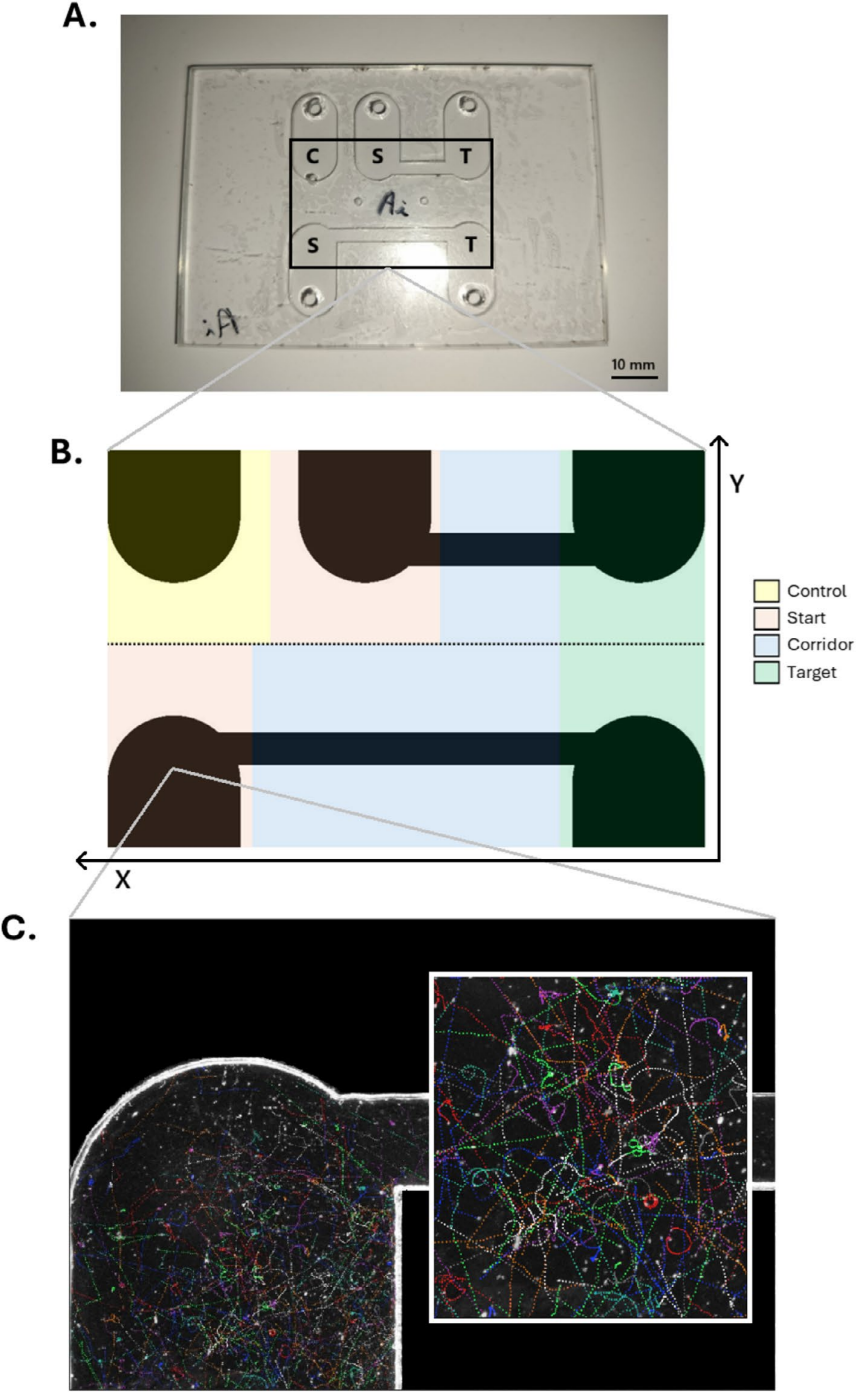
(A) Photograph of a 2D dispersal plate. Distinct plates were used for biological replication, each was identified with a unique coding letter (here ‘Ai’). Three independent landscapes were created: one isolated ‘Control’ (C) patch, and a pair of landscapes each comprising a ‘Start’ (S) and ‘Target’ (T) patches connected by a dispersal corridor (‘Short’ or ‘Long’). The black frame represents the camera field of view, capturing roughly half of each patch and the entirety of both corridors. (B) Image analysis assigns cells to one of four possible ‘Zones’ depending on their X and Y coordinates (see colored legend). The horizontal dotted line denotes the separation between the ‘Short’ (upper) and ‘Long’ (lower) landscapes. The same set of coordinates was used for all pictures. (C) Example picture depicting reconstructed trajectories of individual *Tetrahymena* cells inside the ‘Start’ patch (enlarged on the right), based on a 10‐s picture series (75 frames). The last frame of the temporal sequence is depicted, thus each cell is displayed at the final position of its trajectory.

To allow for continuous tracking of cells throughout the landscape, a novel category of dispersal microcosms was conceived, hereafter referred to as two‐dimensional or ‘2D’ plates (Figure 1). These were designed to mimic the functioning of tube‐based microcosms used in prior studies, allowing ciliates to travel between two distinct habitat patches by crossing a narrow corridor (e.g., Fjerdingstad et al. 2007; Fellous et al. 2011; Pennekamp et al. 2014), though their depth (Z‐axis) was restricted to a shallow water column that allows cells to remain in focus, continuously visible for imaging. To achieve such a design, each 2D plate was assembled from three components: a laser cut 0.8 mm thick plastic layer defining the shape of the microcosm (clear PMMA, Plexiglass), sandwiched between two microscope glass slides (75 × 50 mm, 1.0 mm thick Corning Glass Slides, Large, n°26005 from Ted Pella Inc.). Holes were drilled into the top glass layer, including entry points to allow for culture medium and cells to be introduced into the landscape, and exit points to ensure a flow path for air and liquid to exit. The plastic layer was cut to shape (designed on the OpenScad software, see file in data repository) using a CO2 laser‐cutting machine (ML‐960, https://www.mllaser‐co2.com) and glued to both glass slides using UV glue (LOXEAL UV30‐37). UV light was aimed at the plate for 1 min until the glue had solidified. Lastly, to ensure that the glue was fully cured, plates were left overnight inside an opaque box providing continuous UV light emission.

For the purpose of this experiment, each 2D plate featured three independent landscapes consisting of habitat patches (16 mm long, 10 mm wide planes with a 100 μL capacity) and corridors. The ‘Control’ landscape consisted of one isolated habitat patch. The other two landscapes included one ‘Start’ patch and one ‘Target’ patch, connected by a short (2.5 × 10 mm) or long (2.5 × 20 mm) corridor. Dimensions of the corridors and habitat patches were chosen to respect three constraints: the set of three landscapes must fit within the length and width of the 75 × 50 mm glass slide (with a few millimeters to spare for gluing), most of it should appear within the 24 × 36 mm field of view captured by an overhead camera, and the long corridor must be precisely twice as long as the short corridor (Figure 1).

### 2.3 Dispersal Assays

The experiment consisted in 6.5‐h dispersal assays during which morphology and movement of individual *T. thermophila* cells were recorded for 10 s every 12 min. Preliminary assays were run to help determine total time duration as well as those intervals; the final shooting cycle was scheduled at 396 min to allow for slower strains to approach a dispersal plateau in both fragmentation levels. Data acquisition was performed using an internally‐developed imaging platform, dubbed APIANE (Automated Platform for Imaging of Abundance and Natural Emigration). This setup was previously used for acquisition of morphology and movement data in *T. thermophila* and *Colpidium striatum* (de Bruin et al. 2024) and further improved for the current experiment. APIANE combines darkfield image acquisition equipment with a mobile support for 2D plates or counting slides placed on an active antivibration table (Thorlabs PFA active frame and Nexus Breadboard); the entire setup is automated, controlled by a custom Arduino‐based electronic module. The image acquisition equipment consists of a camera (Sony A7Rv camera equipped with an FE 90 mm F2.8 Macro G OSS lens) and a 208 mm annular led light (CCS LDR2‐208SW2‐LA with DF‐LDR‐208LA diffuser) placed below the 2D plate to create conditions for darkfield imaging; lighting only activated during shooting. The overhead camera was fixed as low as possible for the plate to be in focus, resulting in a near 1:1 magnification: the 24 × 36 mm field of view captures half of each patch and the entirety of both corridors, with 1 pixel covering a 4 × 4 μm^2^ area (Figure 1).

Each of the five strains was replicated on six independent 2D plates and assessed on separate days. This choice was made because the highest source of undesired variation between replicates lies in the culture well and the 2D plate rather than time of the year, owing to a high level of control in experimental conditions and setup. To initiate a dispersal assay, the six plates (replicates of one strain) were ordered randomly and handled under a laminar flow cabinet exclusively used to manipulate *T. thermophila* cells in axenic conditions (Lamsystems Savvy SL). Plates were filled with sterile PPYE medium introduced via the entry points. Cell density in the stock culture was quantified using a correspondence model predicting density from absorbance at 550 nm, measured using a Synergy H1 BioTek plate reader (Jacob et al. 2018; Laurent et al. 2020). The appropriate volume to be inoculated was calculated from the mean of the stock's six independent wells, aiming for ca. 1000 cells per landscape; the same volume was used for simultaneous replicates of a given strain. Cells were inoculated in the ‘Control’ patch, ‘Start’ patch of the ‘Short’ landscape, and ‘Start’ patch of the ‘Long’ landscape, always in this order. Then, a narrow band of sterile adhesive polyethylene film (Titer Tops Z688630, not permeable to gas) was used to seal entry and exit holes, preventing evaporation and maintaining sterility of the medium during the experiment. Plates were inoculated and sealed sequentially with a 2‐min interval, then carefully moved onto the mobile support.

APIANE was parameterized to automatically image the same 2D plate every 12 min, cycling from one plate to the next every 2 min (32 cycles, from 24 to 396 min; before 24 min, cells had not yet reached the camera field of view). For each imaging session, a 10 s series of greyscale pictures was shot (10 s burst shot at 7.5 FPS = 75 frames, shutter speed = 1/160 s, aperture = F11, ISO = 6400). At the end of the dispersal assay, 2D plates were checked under a macroscope to confirm that cells had dispersed from the ‘Start’ to the ‘Target’ patch and verify the absence of anomalies (such as leaks or evaporation). Plates were thoroughly rinsed by passing distilled water through the landscapes and dried using compressed air.

### 2.4 Estimating Response Variables Using Image Analysis

To estimate cell abundance, morphology and movement traits of individual cells, we used an improved version of image analysis methods previously developed by our team (Pennekamp and Schtickzelle 2013; Pennekamp et al. 2015), which has since gained traction in protist ecology (Giometto et al. 2014; Pennekamp et al. 2019; Moerman et al. 2020; Suryanto et al. 2022; Thierry et al. 2025). Image analysis using Fiji (Schindelin et al. 2012) was incorporated into Python 3 scripts (van Rossum and Drake 2014) to automatically process each sample recorded with APIANE. The first step (‘grouping’) selected and grouped all pictures belonging to a given sample based on their timestamp, thereby verifying that 75 pictures were recorded. The second step (‘particle analysis’) involved removing the scene background and areas of the plate located outside the carved out landscapes to facilitate image processing (for a detailed explanation, see Figure S1 in Appendix S2). The Fiji ‘particle analysis’ algorithm was then applied separately for each frame, to identify and characterize all particles fitting the constraints of a set of parameters including gray scale values (to discriminate between white particles and a black background) and size range (surface area in pixels) corresponding to *T. thermophila* cells. The third step (‘tracking’) consisted in linking particles identified as *Tetrahymena* across consecutive frames to reconstruct individual movement trajectories (Figure 1) based on maximum distance that a cell can travel between frames, allowing to discriminate ciliates from moving artifacts (particles that may appear moving due to liquid motion generated by neighboring cells) and avoid linking cells that were in fact different individuals. For each reconstructed trajectory, swimming speed (μm s^−1^) was computed as the gross displacement achieved over the movement duration, and trajectory linearity as the net displacement (i.e., Euclidean distance between the start and end positions of the trajectory; Buskey 1984) divided by the gross displacement. Further data cleaning of trajectories not matching quality criteria (e.g., minimum values for displacement, trajectory duration and cell detection frequency) was performed using R version 4.4.1 (R Core Team 2021). For a complete list of analysis parameters and post‐processing quality criteria used during the main steps of image processing, see Table S2 in Appendix S3.

Several variables were extracted from the set of trajectories identified in each sample (i.e., a series of 75 pictures shot on one 2D plate at a single time point). Cell abundance was defined as the mean number of trajectories detected on each frame in a given location (i.e., either the ‘Control’ patch, or any distinct area of the short and long landscapes). Dispersal rate (i.e., abundance in the ‘Target’ patch, divided by total abundance across the ‘Corridor’, ‘Start’ and ‘Target’) was computed within short and long landscapes and plotted over time (from *t* = 24 min to *t* = 396 min). Dispersal rate at termination was computed as the median of the five latest values measured in a landscape, and time until half dispersal as the first time point at which half of that median was exceeded. At the individual level, movement traits included swimming speed (μm s^−1^) and trajectory linearity (higher meaning ‘straighter’; corrected for trajectory duration, see Appendix S4). Morphology traits included cell size (area in μm^2^) and cell shape (ratio of the major and minor axes of a fitted ellipse, smaller meaning ‘rounder’).

Morphology and movement variables were distinguished on the basis of *Strain* identity, *Fragmentation* level (‘Short’, ‘Long’), *Zone* (‘Start’, ‘Corridor’, ‘Target’) and *Plate* (i.e., replicate, out of six distinct plates identified by a coding letter). Individual cells were assigned a level for *Zone* and *Fragmentation* on the basis of their X and Y coordinates observed at each frame (see Figure 1). To ensure equal sample size and analysis at the appropriate replication level, phenotypic traits were first averaged over each trajectory (the individual level), then over all trajectories identified in a given zone (population level), for each time point; a data filter was applied to the ‘Corridor’ and ‘Target’ patches, so that only time points that showed at least five individual trajectories in those zones would be considered. Further, to limit the interpretation of data to what would count as the ‘early’ phases of dispersal, a narrower subset of data was created which only included time points from *t* = 24 min (start of shooting) to each population's time until half dispersal. For graphical depictions and statistical analyses, values of phenotypic traits were averaged across all time points included under these criteria.

To test specific hypotheses concerning movement decisions, the shape of dispersal curves and expected distribution of movement traits were computed using an individual‐based simulation model running on NetLogo software version 6.3.0 (Wilensky 1999). Individual displacement was simulated under the null hypothesis that cells move only according to their recorded speed and linearity values (as observed in the ‘Control’ patch across all time points of the experiment), do not adjust their movement behavior as time progresses, and do not take individual decisions based on interactions with conspecifics or their current position in the landscape (such as U‐turn movements when entering the corridor, indicating that cells refrain from emigrating). For a full description of this model, see Appendix S5. Three additional variables were conceived to compare experimental data with simulated outputs lacking decision rules. Emigration rate was computed as the summed abundance in the ‘Corridor’ and ‘Target’, divided by total abundance. Besides, it was observed through preliminary recordings that the expansion front of inoculated cells progresses through the ‘Start’ patch at varying rates between strains (F. Manzi, personal observation), potentially resulting from aggregative behavior between conspecifics (Chaine et al. 2010; Jacob et al. 2016). To quantify this gradual displacement, we measured height of the front (90% quantile of the Y coordinate in the ‘Start’ patch, corresponding to the height above which only 10% of cells were located), height of the rear (10% quantile, corresponding to the height above which 90% of cells were located), and compared their progression over time between experimental data and simulated outputs.

### 2.5 Statistical Analyses

Data were analyzed using R version 4.4.1 (R Core Team 2021). Response variables characterizing the shape of dispersal curves (dispersal rate at termination, time until half dispersal) were analyzed via a mixed and crossed two‐way ANOVA with *Fragmentation* as a fixed factor, *Strain* as a random factor and their interaction (random) using the ‘EMSanova’ function in the ‘EMSaov’ package (Choe et al. 2017). Assumptions of normality and homoscedasticity of the residuals were verified by visual inspection of quantile‐quantile plots, residuals against fitted values and distribution of the residuals. Pairwise comparisons between *Fragmentation* levels within each *Strain* were run using Tukey's HSD test (‘TukeyHSD’ function in base R). Movement and morphology traits were analyzed via a three‐way ANOVA with *Zone* and *Fragmentation* as fixed factors, *Strain* as a random factor, and all interactions. In these analyses, *Strain* was considered a random factor as those used for the study were randomly chosen among a larger set of available strains; they play the role of biological replicates to test for intraspecific variation in the responses of interest. Error terms for *F*‐tests were computed as described in Sokal and Rohlf (1995) for both ANOVAs. Two‐sample Kolmogorov–Smirnov tests were used to compare data distribution of movement traits across *Zones* at discrete time points (function ‘ks. test’ in the ‘KSgeneral’ package; Dimitrova et al. 2020).

## 3 Results

### 3.1 Dispersal‐Related Traits

**FIGURE 2:**
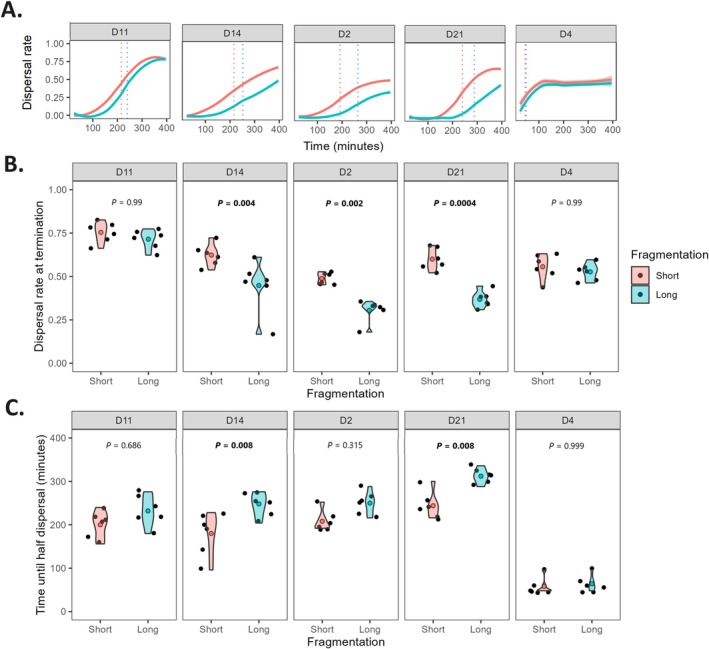
(A) Dispersal rate over time (LOESS smoothing on 32 time points over 396 min) for one representative replicate of each *T. thermophila* strain, at two levels of landscape *Fragmentation*. The depicted populations all ran on plate ‘Gi’ (for curves of all replicates, see Figure S4 in Appendix S6). Colored dotted lines denote, for each *Fragmentation* level, the first time point at which half of the population's final dispersal rate was exceeded. (B) Dispersal rate at termination. (C) Time until half dispersal. For each strain, (B, C) show a violin plot of six independent populations in black with their mean value in red (‘Short’) or blue (‘Long’). *p*‐values denote Tukey's HSD post hoc contrasts for the *Fragmentation* effect.

Dispersal rate through time followed a sigmoid curve in most populations (Figure S4 in Appendix S6), with a generally slower increase and lower dispersal rate at termination in longer landscapes (Figure 2A). Dispersal rate at termination was significantly impacted by corridor length (*F*_1;4_ = 10.38, *p* = 0.032), with some variation among strains (*Fragmentation* × *Strain* interaction: *F*_4;50_ = 4.82, *p* = 0.002; for detailed ANOVA results, see Table S3 in Appendix S6). Three out of five strains reached a significantly lower dispersal rate in the long landscape, with a difference of up to 0.25; in the two other strains, dispersal converged before the end of the experiment (Figure 2B). On average, most strains took a longer time to reach half dispersal with a long corridor, but the magnitude of this difference (up to 70 more min) differed among strains (Figure 2C); it was especially small in strain D4, which dispersed much faster than any other strain (*Fragmentation*: *F*_1;4_ = 13.62, *p* = 0.021; *Strain*: *F*_4;50_ = 91.58, *p* < 0.0001; *Fragmentation* × *Strain* interaction: *F*_4;50_ = 2.35, *p* = 0.067; for detailed ANOVA results, see Table S3 in Appendix S6).

**FIGURE 3:**
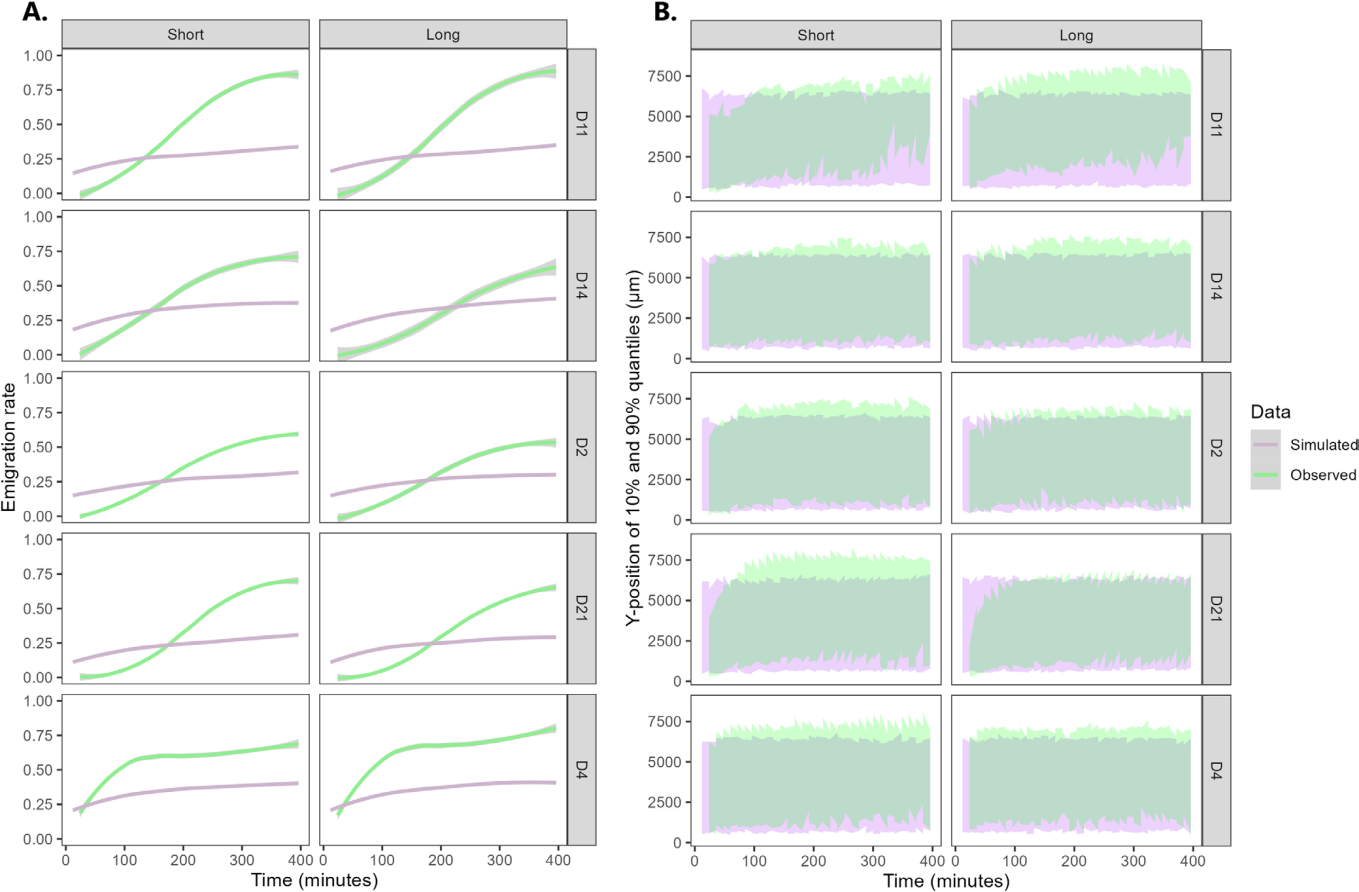
Comparing temporal dynamics of experimental data (‘Observed’) with outputs from a null movement model (‘Simulated’). (A) Emigration rate (proportion of cells in the ‘Corridor’ plus ‘Target’ zones). (B) Population's front (90% quantile of Y‐coordinate along the height of the ‘Start’ patch) and rear (10% quantile) margins. Data smoothing was applied using the LOESS method on pooled data from six replicates for each *Strain* × *Fragmentation* combination.

During the early stages of dispersal assays, emigration rate was consistently higher in simulated outputs than in experimental populations (Figure 3A). With the exception of the faster dispersing strain D4, observed emigration was still null at the start of shooting (*t* = 24), whereas simulated populations had already reached a 20% emigration rate from the ‘Start’ patch. This head start was maintained for at least 120 min after initiating dispersal assays; past this point, experimental populations had caught up with simulated outputs, eventually reaching much higher emigration rates than simulated by the model. Similarly to emigration rate, the positioning of resident cells along the height of the ‘Start’ patch began with a head start in simulated outputs compared to observed data (Figure 3B). Over time, both the 10% quantiles and 90% quantiles of experimental populations' Y‐coordinate increased and surpassed that of simulated outputs, indicating that both the rear and the front of starting populations had progressed upward from their point of entry. Contrary to this pattern, the 10% and 90% quantiles of simulated populations remained constant within the ‘Start’ patch.

### 3.2 Movement and Morphology

**FIGURE 4:**
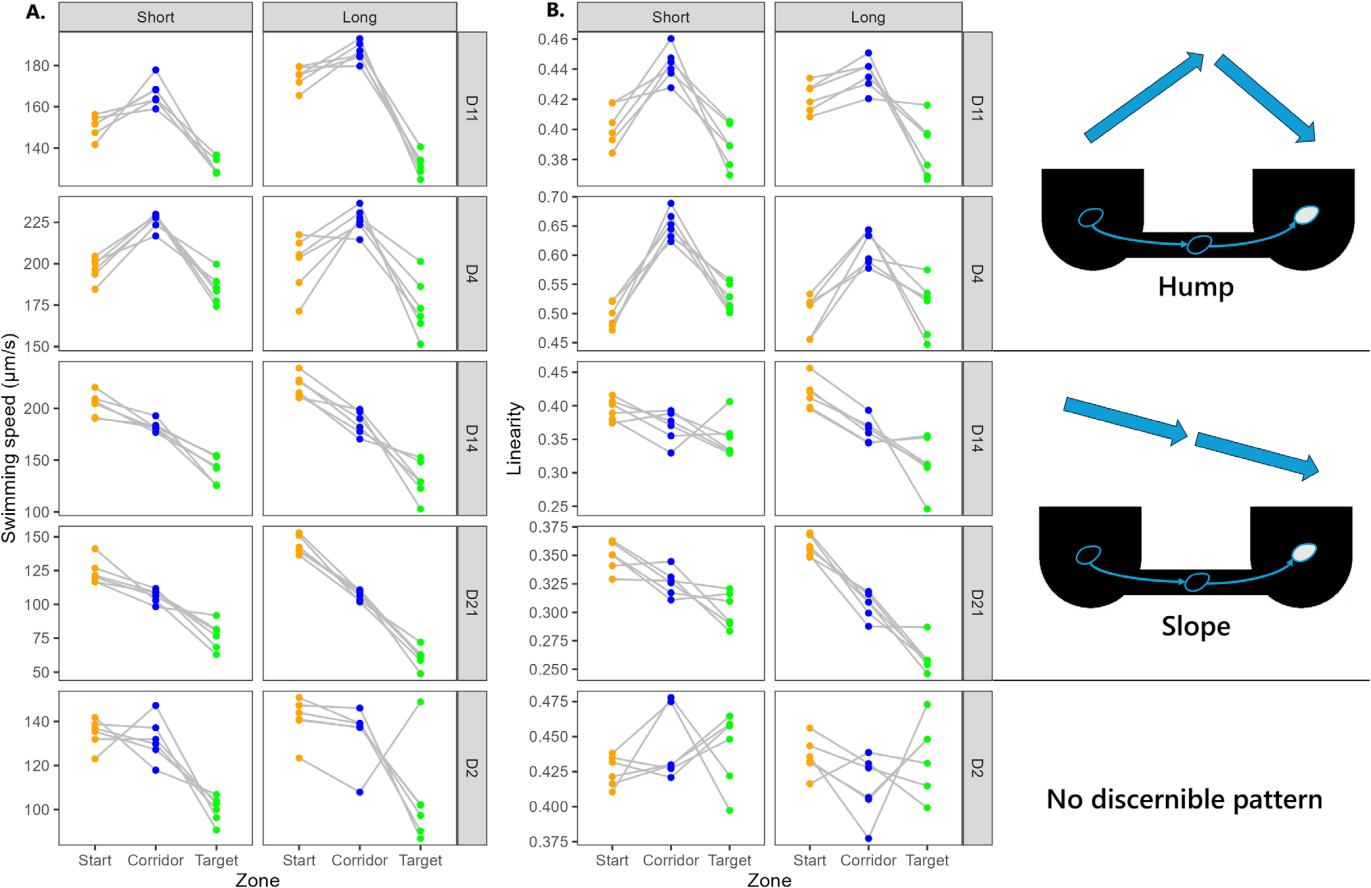
Cell movement differed across the ‘Start’, ‘Corridor’, and ‘Target’ zones in both swimming speed (A) and trajectory linearity (B). Depicted values are averaged across all time points from *t* = 24 min (start of shooting) to each population's respective time until half dispersal. For easier visualization, data were centred by landscape (i.e., for each data point, the mean of the *Plate* within its respective *Strain* × *Fragmentation* level was subtracted, before adding the mean of all plates in this level). Dots belonging to the same landscape are connected. Strains were grouped based on similar shifts in movement traits displayed across the landscape: ‘hump’ (increase at emigration, decrease at immigration), ‘slope’ (consecutive decrease at emigration and immigration), or no discernible pattern.

Movement of cells differed in the three zones (‘Start’, ‘Corridor’ and ‘Target’), with some variation among strains (Figure 4; for detailed ANOVA results, see Table S4 in Appendix S6). Two strains (D4 and D11) displayed higher swimming speed and linearity in the ‘Corridor’ compared with either the ‘Start’ or ‘Target’ patch, which was described as a ‘hump’ pattern (i.e., increase at emigration, followed by a decrease at immigration); combined, these two parameters indicate that cells traveled the corridor quite faster than the habitat patches. Two other strains (D14 and D21) showed a consecutive diminution in speed and linearity after emigration, then immigration, which was described as a ‘slope’ pattern. The remaining strain (D2) showed no discernible pattern and greater variability in both movement traits among its replicates. Across those three groups, the general observation remains that swimming speed consistently decreased at immigration, with only one exception (replicate ‘Ai’ of strain D2).

**FIGURE 5:**
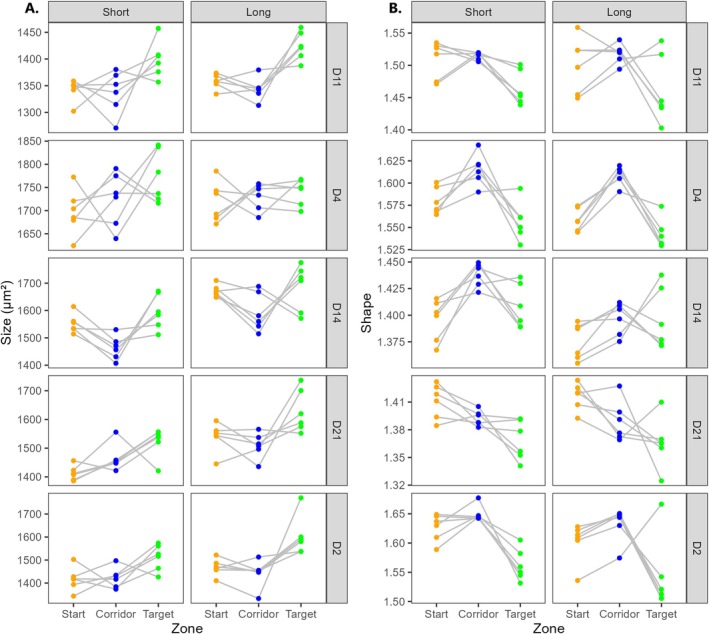
Cell morphology differed across the ‘Start’, ‘Corridor’, and ‘Target’ zones in both cell size (A) and cell shape (B). Depicted values are averaged across all time points from *t* = 24 min (start of shooting) to each population's respective time until half dispersal. For easier visualization, data were centred by landscape (i.e., for each data point, the mean of the *Plate* within its respective *Strain* × *Fragmentation* level was subtracted, before adding the mean of all plates in this level). Dots belonging to the same landscape are connected.

Similarly, morphology differed in the three zones, with some variation among strains (Figure 5; for detailed ANOVA results, see Table S4 in Appendix S6). Variation among replicates was larger for morphology than for movement traits, encompassing both the absolute values of response variables within a zone and the direction of phenotypic shifts between zones. In a large number of experimental populations, cell size was highest in the ‘Target’ patch, showing up to a 15% increase at immigration (compared with the ‘Corridor’); by contrast, patterns of phenotypic shifts at emigration were much less discernible. With regards to cell shape, three out of five strains (D2 and D4, as well as D14 in ‘Short’ landscapes only) displayed a similar ‘hump’ pattern as was described for swimming speed and linearity, indicative of increased cell elongation at emigration, followed by a return to similar or lower values in the ‘Target’ than were observed in the ‘Start’ patch.

### 3.3 Temporal Shifts in the Distribution of Movement Traits Across the Landscape

**FIGURE 6:**
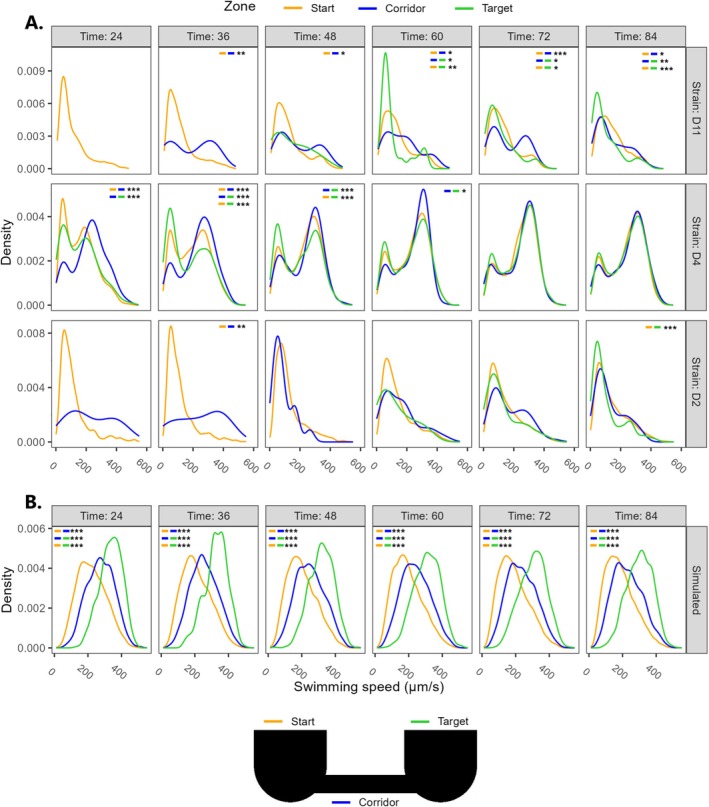
(A) Observed distribution of swimming speed over the first six time points of the experiment for strains D11, D4 (‘hump’ pattern) and D2 (no discernible pattern). (B) Simulated distributions were generated by the null model (data from all five ‘simulated’ strains were pooled). For both observed and simulated values, only data from the ‘Short’ fragmentation level are depicted. *p*‐values provided by Kolmogorov–Smirnov tests are depicted at the top of each panel, each comparing data distribution between paired zones of the landscape (see colored legend). Only significant comparisons are reported on the figure: *** (*p* ≤ 0.001); ** (*p* ≤ 0.01); * (*p* ≤ 0.05).

To shed further light on the characteristic ‘hump’ pattern in swimming speed displayed by strains D11 and D4, further analyses were conducted at the individual level. Specifically, it was asked whether this phenomenon could be detected in ‘pioneer’ cells (i.e., those which were first observed to travel the ‘Corridor’ and reach the ‘Target’ patch), which would imply an early emergence of dispersal syndromes across distinct zones of the landscape. For this purpose, swimming speed distribution was visualized across six consecutive time points, starting from *t* = 24 min (Figure 6A); strain D2 was added due to its replicates behaving halfway between the ‘hump’ and ‘slope’ patterns. Starting from the earliest time point at which emigrating cells were detected (either *t* = 24 min or *t* = 36 min), all three strains showed a skewed distribution towards higher swimming speed values in the ‘Corridor’. Disperser cells were first detected in the ‘Target’ patch with varying timing, which was fastest in D4 (*t* = 24 min) and slowest in D2 (*t* = 60 min). The distribution of swimming speed in the ‘Target’ patch was skewed towards lower values than the ‘Corridor’, comparable to that of the ‘Start’ patch. Neither the ‘Corridor’ nor ‘Target’ zones showed values for swimming speed outside the range of the ‘Start’ patch (e.g., [20–500 μm/s] for strain D4). To complement this visualization, swimming speed distribution in the three zones was additionally depicted using a color gradient for six consecutive time points (*t* = 24 min onward) and compared to that of the isolated ‘Control’ patch, which does not permit cell emigration and thus spatial segregation outside of its boundaries (see Figure S5 in Appendix S6). Distribution in the ‘Control’ landscape and ‘Start’ patch of the ‘Short’ landscape remained nearly identical for a given strain: starting with a peak at low velocity at the start of shooting (*t* = 24 min), data was later skewed towards higher swimming speed values, with a varying magnitude across strains (an extreme example being displayed by strain D4). This indicates that similar shifts in the distribution of phenotypic traits can emerge over time in both locations, even in the absence of cell departure.

In simulated outputs, disperser cells were recorded in the ‘Target’ patch as early as *t* = 12 min (an additional time point recorded one cycle ahead of real dispersal assays); this was true for all strains and at both fragmentation levels. Simulated data displayed a striking tripartite distribution that remained constant throughout the six earliest time points: swimming speed distribution overlapped in the three zones, with a skew towards lower velocity in the ‘Start’ and higher velocity in the ‘Target’ patches (Figure 6B). Overall, swimming speed in the ‘Start’ and ‘Corridor’ showed similar distributions between experimental and simulated data, whereas data in the ‘Target’ was skewed in opposite directions between both data types. As opposed to swimming speed, the previously described ‘hump’ pattern relative to trajectory linearity could only be detected in strain D4 within the first six time points for the experiment (see Figure S6 in Appendix S6). As early as *t* = 24 min, that strain displayed a very strong bias towards increased linearity in the ‘Corridor’ compared with both habitat patches. The observed distribution for D4 directly contradicts simulated outputs on all strains, which rather predict an extreme bias towards higher linearity in the ‘Target’ patch (albeit no difference between ‘Start’ and ‘Corridor’).

## 4 Discussion

To understand how individual movement decisions translate to dispersal events at the population scale, there is a need for in situ observation of trajectories across all stages of the dispersal process (including transience in between habitat patches). Adding to the convenience of artificial microcosms, the association of an automated shooting platform with ‘two‐dimensional’ dispersal landscapes has enabled the collection of invaluable data that had eluded most previous studies on *Tetrahymena*. This includes (i) data collection of morphology and behavior inside dispersal corridors, which now permits direct comparisons of traits at emigration (comparing ‘Start’ and ‘Corridor’) and immigration (comparing ‘Corridor’ and ‘Target’); (ii) a progressive assessment of dispersal rate at numerous time points throughout dispersal assays; (iii) visualizing shifts in the distribution of phenotypic traits over time, allowing comparisons between pioneer cells (i.e., the first to enter the corridor or target patches) and later dispersers.

### 4.1 Dispersal, Emigration and Spatial Use of the Landscape

Genotypic variation in dispersal propensity has long been reported in *T. thermophila* . On that note, all strains used in the present study had previously been employed in two‐patch dispersal assays, which either lasted a similar duration (Pennekamp et al. 2014, 2019; Junker et al. 2021; Campana et al. 2022; Cayuela et al. 2022) or a much longer seventeen hours (Fjerdingstad et al. 2007). Dispersal rates recorded after 396 min ranged from 0.5 to 0.75 in ‘Short’ two‐dimensional landscapes, which was comparable to the usual scope of values reported in tube‐based assays. This points towards validating those microcosms as effective dispersal landscapes, which generate patterns of movement between patches that differ from simple diffusion across the landscape, thus enhancing comparability of our data with the substantial body of work generated since Fjerdingstad et al. (2007). Some differences emerge in our findings, such as D21 showing lower dispersal rates than expected in the ‘Long’ landscapes, compared with previous studies in which this strain was used; strain D2 also behaved quite differently in 2D landscapes, displaying comparably low dispersal rates across both fragmentation levels (Pennekamp et al. 2014; Junker et al. 2021).

As previously mentioned, an advantage of 2D plates over previous studies resides in the ability to visualize dispersal rate at any time point of an assay, aside from its termination. The general shape of dispersal curves proved similar across a majority of strains and fragmentation levels: most populations showed a roughly sigmoid (S‐shaped) curve, revealing the existence of an early lag phase prior to instigating large scale dispersal movements across the landscape. Assimilating the cells' sudden transfer from stock cultures to the constrained space of 2D plates as a trigger for behavioral plasticity, then such a lagging phase can be expected. Plastic changes can be characterized by a time lag between their onset and their observation, corresponding to the mandatory steps of cue detection and information transduction (e.g., altered transcriptional activity) that lead to the expression of behavioral changes (Bukhari et al. 2017; Dupont et al. 2024). A notable exception to this pattern was strain D4, which showed closer to a logarithmic curve, indicating a very early saturation of cell abundance in the ‘Target’ patch. The presence of a lagging phase in most strains apart from D4 is further clarified when comparing emigration rates (i.e., exits from the ‘Start’ patch) between experimental and simulated data. Indeed, most strains took over 2 h to surpass the initial emigration rate displayed by simulated cells, indicating that part of this lagging pattern was already generated at the emigration stage. In the null movement model, cell displacement was only determined by each strain's inherent tendency for slow or fast (‘swimming speed’) and more or less tortuous movements (‘standard deviation of turning angles’). Therefore, any major deviation between experimental data and simulated outputs should point towards additional parameters that control emigration decisions, including propensity for dispersal, landscape configuration, resource levels, and/or the proximity of conspecifics. Here, most strains seemingly shared an initial reluctance to leave the ‘Start’ patch; by comparison, D4 appeared to be much less driven by such behavioral barriers to emigration, the exact nature of which would need to be determined by more targeted studies investigating cell attraction or the presence of chemical cues within those landscapes.

Significant differences in the time until half dispersal indicate that strains differed in the pace at which they progressed through the landscape, regardless of their dispersal rate at termination. For instance, strain D4 had already saturated at a rate of ~0.50 in under 100 min, whereas strain D2 required nearly 400 min to reach a similar plateau. Interestingly, strain D4 showed by far the highest movement speed in the ‘Corridor’, and also exhibited the straightest movements across all three zones (Figure 4). Spatial localization of its front and rear margins within the ‘Start’ patch was comparably higher than other strains at the start of shooting, suggesting that D4 cells were also faster at reaching the corridor's entrance after being inoculated near the opposite end of the patch (Figure 3B). Adding to the prior topic of emigration decisions, these observations confirm that D4 quickly spread through both dimensions of the microcosm, the combination of which resulting in exceptionally fast‐paced colonization of the landscape. Overall, this strain's movement behavior seemed consistent with theoretical predictions from the literature: faster individuals are expected to reach range margins faster than their conspecifics (Fronhofer and Altermatt 2015), whereas swimming speed and trajectory linearity were both shown to positively correlate with dispersal in three‐dimensional landscapes (Fjerdingstad et al. 2007; Schtickzelle et al. 2009; Pennekamp et al. 2014), explaining up to 47% of genetic variation in the dispersal rate of *T. thermophila* (Pennekamp et al. 2019). Our results also provide some nuance to these predictions: such movement traits may better predict the rapidity at which a strain's theoretical maximum for dispersal is attained in a two‐patch landscape, rather than the value of this maximum itself; this is evidenced by slower swimming strains such as D11 and D21 having reached higher dispersal rates at termination than D4.

Contrary to our expectations, emigration rates were sensibly similar between ‘Short’ and ‘Long’ landscapes. Although this was expected for simulated outputs (which lacked decision rules), we hypothesized a stronger reluctance to emigrate in the ‘Long’ corridor, manifesting as more frequent U‐turns after crossing its boundary. U‐turn movements can be expected in fragmented landscapes: dispersers from many taxonomical groups tend to perform explorative movements, which are useful for gathering information about biotic and abiotic parameters of the landscape without fully committing to dispersal (Doligez et al. 2004; Clobert et al. 2009; Bocedi et al. 2012); a similar behavior was sometimes observed during preliminary assays (F. Manzi, personal observation). Moreover, the perceived contrast between habitat patches and their surrounding environment can reduce an individual's propensity to cross its habitat borders; this has been observed in natural populations of several butterfly species (Ries and Debinski 2001; Schtickzelle et al. 2006; Conradt and Roper 2006), translating to a strong return tendency (i.e., U‐turns) performed at habitat patch boundaries by the bog fritillary butterfly (Schtickzelle and Baguette 2003). This was not corroborated by emigration rates in our study, indicating that at any given time point, the same proportion of cells had exited the ‘Start’ patch in both fragmentation levels. This implies that the detected fragmentation effects on dispersal pace and rate may simply derive from cells requiring more time to complete transience as inter‐patch distance increases (see Figure 2).

Interestingly, both the front (90% quantile) and rear (10% quantile) of experimental populations appeared to be moving conjointly, as if one cohesive but dynamic cluster of individuals displaced itself along the Y‐axis of the ‘Start’ patch (away from the inoculation point). Whether or not cells located at the moving front of those clusters bore a higher dispersal ability could not be verified from the present data (however, it is predicted in the literature; Phillips 2016). Social aggregation is a known phenomenon in *T. thermophila* , which shows genetic variability and has been studied under similar experimental setups (Schtickzelle et al. 2009; Chaine et al. 2010; Jacob et al. 2015). *Tetrahymena* ciliates are capable of producing hormones such as EGF (epidermal growth factor; Csaba et al. 2004), which play a role in cell division and survival (Kristiansen et al. 1996; Selivanova et al. 2002), and aggregation is thought to favor the exchange of similar molecules under harsh conditions (Christensen et al. 1996, 1997). On that note, Jacob et al. (2016) found that dispersers from highly aggregative strains showed significantly reduced colonization efficiency from a single founder cell compared with less aggregative ones. Assuming that propensity for aggregation also varies within monoclonal populations—as it does for many other traits (Fjerdingstad et al. 2007; Cayuela et al. 2022) – it is conceivable that pioneer cells are more successful at colonizing distant patches due to individual preference against clustering behavior.

### 4.2 Swimming Behavior and Cell Morphology Differ Across Distinct Zones of the Landscape

Besides the expected effects of habitat fragmentation on dispersal propensity, prior theoretical and experimental studies have also contemplated the implications of increased fragmentation on the expression and evolution of dispersal syndromes (Cote et al. 2017; Jacob et al. 2020). Here, variations in corridor length (either 10 mm or 20 mm, the same rate of increase which was previously used with *T. thermophila* ; Laurent et al. 2020) only had minor effects on movement and morphology traits, seeing as the aforementioned ‘hump’ and ‘slope’ patterns were greatly conserved across both fragmentation levels. Overall, changes in movement and morphology traits displayed at immigration seemed compatible with cells responding to increased resource levels and a lower density of conspecifics in the ‘Target’ patch. There was a general increase in cell size, which may result from more efficient nutrient acquisition compared with the initially crowded ‘Start’ patch, as well as overall reductions in swimming speed and linearity that are reminiscent of typical foraging movements (ciliates tend to move tortuously in confined regions, matching the limited depth and high density of cells within these microcosms; Chang et al. 2011). Increased time allocation dedicated to foraging can also be expected as dispersers proceed with the steps of transience, then settlement, as was observed in natural populations of the mammal *Suricata suricatta* (Harrison et al. 2021).

Aside from this generalized pattern, two distinct strategies were identified depending on the directional shift of swimming speed and linearity displayed at emigration: strains either followed a ‘hump’ (indicative of increased trait values at emigration followed by a decrease at immigration), or a linear decreasing ‘slope’ (reflecting consecutive diminutions in movement traits at emigration, then immigration). While strains from the ‘hump’ group (D4, D11) exhibited a set of syndromes typically reported in disperser phenotypes (i.e., increased speed and linearity), those phenotypes were only expressed during the step of transience, contrasting with numerous reports of faster and straighter movements in cells sampled from the ‘Target’ patch of terminated dispersal assays (Fjerdingstad et al. 2007; Schtickzelle et al. 2009; Pennekamp et al. 2014; Jacob et al. 2020). The remaining strains tested here also challenged these expectations, especially D14 and D21: those tended to decelerate and reduce their linearity during transience, yet were still able to reach very high dispersal rates, including greater values than D4 from the ‘hump’ group (> 0.5).

Acquiring a varied set of dispersal strategies can enhance a species' ability to cope with environmental conditions that are spatially and temporally heterogeneous (Cheptou et al. 2008); this applies to the natural environment of *T. thermophila* , considering the patchy disposition of abundant food sources, along with seasonal changes in the community composition of emergent macrophytes and primary producers (Doerder and Brunk 2012; Jenačković et al. 2016; Yang et al. 2020). In this context, the ‘hump’ strategy maps rather well onto the colonization dynamics of *T. thermophila* : considering elevated risks of predation and energy exhaustion between habitat patches, linear searching should facilitate locating favorable patches before death occurs during dispersal (Zollner and Lima 1999). Though it is unclear to which extent *T. thermophila* can obtain information about distant feeding spots in the water column, it was also predicted that uninformed search strategies should favor constrained turn angles, i.e., straighter movement (Wilson et al. 2013). The ‘slope’ pattern seems comparably harder to reconcile with dispersal dynamics in patchy environments. Because depleting resources such as nutrients or dissolved oxygen may be more difficult to acquire during transience in the water column, the alternative strategy to slow down may be equally stable from an evolutionary standpoint; by performing less demanding movements over long distances, individuals may be able to limit energy expenditure and thus survive until a suitable patch is located (Brans et al. 2024). Alternatively, the ‘slope’ pattern might not have emerged from a decision‐based strategy, but rather from energy constraints due to more limiting conditions encountered during transience, which would have prevented individuals from maintaining their movement speed. The explanation as to which genotypes associate with either emigration strategy may thus rely on genetically‐determined divergences in resource allocation, but also depend on their respective ability to acquire information about distant patches in nature. Correlating the dispersal strategy exhibited by a larger set of strains with other life history traits seems needed to better explain what drives such intraspecific variation in dispersal strategies.

### 4.3 Disentangling Spatial Sorting From Phenotypic Plasticity

The striking differences in movement and morphology traits observed at emigration and immigration confirmed one of our primary assumptions, being that distinct dispersal syndromes would likely emerge at the landscape scale. Indeed, the literature states that habitat fragmentation can lead to a spatial structuring of local conditions, in turn affecting the expression of syndromes (Cote et al. 2017). Such a phenomenon likely applied to each distinct zone of our bidimensional landscape, which could have shown heterogeneity in oxygen availability and resource levels across its length, owing to structural narrowing along the corridor and the initial lack of conspecifics within the ‘Target’ patch. The present data begs to question whether those patterns reflected a plastic adjustment of dispersal syndromes throughout the experiment, or whether those shifts simply derived from spatial segregation of pre‐existing phenotypes among the starting population. First, our monoclonal setup excluded genetic variation as a possible source of phenotypic differences between dispersers and residents (Jacob et al. 2020; Cayuela et al. 2022). Individual cells of *T. thermophila* typically live for a few hours in suboptimal laboratory conditions, with an estimated generation time of 17 h at 23°C (Chaine et al. 2010; Jacob et al. 2015), which also precludes a major role of novel genetic variation (i.e., mutations). Nevertheless, intraspecific trait variation can be driven by non‐genetic mechanisms in protists (Raffard et al. 2024), thus spatial sorting based on standing variation remains plausible. Within‐strain variability in morphology and behavior can emerge due to a variety of causes including cell age (i.e., time since last division) or distinct developmental noise (i.e., ontogeny) resulting from individual differences in resource acquisition, physical damages or encounters with foreign particles and conspecifics. Another source of variation put forward by Cayuela et al. (2022) derives from the unusual ability of the somatic nucleus to divide amitotically, which can result in different chromosomal copy numbers between cells of a clonal population despite no changes in DNA sequences (Verdonck et al. 2022). Copy number variation in ciliates seems to be higher for chromosomes carrying environmental response genes over housekeeping genes, and partial ploidy changes in *T. thermophila* happen to be reversible upon relief from environmental stress (Cheng et al. 2020; de Francisco et al. 2018).

Here, the characteristic ‘hump’ pattern displayed by strains D4 and D11 suggested at least some level of reversible plasticity in morphology and movement traits across the landscape. On the one hand, a positive shift in speed at emigration could be explained by the aforementioned sorting hypothesis: cells that already displayed an elevated velocity in the ‘Start’ patch emigrated first, then maintained that phenotype during transience. This is especially plausible, considering that simulations (which permitted no adjustment of velocity across zones of the landscape) generated similar distributions in the corridor as did experimental data, showing a bias towards higher swimming speed. On the other hand, mean values for both movement traits decreased again at immigration—in the case of swimming speed, reaching lower averages in the ‘Target’ than within the ‘Start’ patch—which seems hardly compatible with strict cell sorting. This would imply that among cells in the corridor, slow individuals immigrated in majority while faster cells either stayed in the corridor or returned to the ‘Start’ patch; besides being difficult to explain from a biological standpoint, such a process would also be opposite to what simulated outputs predicted. A second argument against spatial sorting derives from similar distributions of movement traits between the ‘Start’ patch and the isolated ‘Control’ landscape. Since the ‘Control’ did not allow exits from its boundaries (only temporary absence from the camera field of view), temporal shifts in speed distribution within this patch must already result from phenotypic plasticity, potentially attributed to cells adjusting to a novel environment after being transferred from stock cultures. Then, the fact that speed distribution within the ‘Start’ patch did not deviate from the ‘Control’ suggests that supposedly faster cells exiting the patch would not have a noticeable impact on speed distribution within starting populations, at least throughout the first six time points of dispersal assays (see Appendix S6). This is reinforced by the fact that trait distribution between the ‘Start’ and ‘Control’ patches remained similar until the final time point of the experiment (data not shown).

Given the nature of our data, a more convincing hypothesis for distribution shifts at immigration consists of plastic adjustments in movement behavior, which may have occurred after dispersers had already settled in the ‘Target’ patch. Providing credit to this interpretation, we observed a lag between the first time point at which cells were detected in the ‘Target’ and the moment when swimming speed distribution in this patch differed statistically from that of the ‘Start’ or ‘Corridor’ (see for instance strains D11 and D2 in the ‘Short’ landscape). Thus, experimental data suggest that early immigrants did not necessarily differ from other cells in the corridor: instead, given a brief time lag following their arrival in the ‘Target’ patch, those cells appeared to plastically adjust their behavior towards slower movement. As mentioned prior, such a time lag in the expression of phenotypic changes is not unforeseen and can easily be explained by incompressible time requirements imposed by the underlying mechanisms of phenotypic plasticity (Dupont et al. 2024). Besides, dispersal movements are predicted to result in such plastic changes, for instance due to differing allocation strategies between habitat patches that differ in environmental conditions (e.g., resource levels), as well as energy expenditure and specific movement strategies associated with costs and risks incurred during dispersal (Bonte et al. 2012; Winandy et al. 2019).

In a succession of tube‐based assays investigating the transgenerational plasticity of dispersal syndromes in *T. thermophila* , Cayuela et al. (2022) concluded that morphological and movement traits associated with dispersal are indeed plastic, and can be inherited over successive generations. Genotypic identity also seemed to affect the reversibility of plastic changes in that study, which coincides with our observation of two distinct genotypic groups that seemed to either reverse (‘hump’) or accentuate (‘slope’) certain shifts in movement traits displayed across the length of the landscape. Despite such hints at reversible plasticity, the collected data also suggest some limits to the degree of plasticity that can be displayed by *T. thermophila* in fragmented landscapes. Here, swimming speed values recorded in the ‘Target’ and ‘Corridor’ fell within a range that pre‐existed in the starting population. This shows that neither at emigration nor immigration did *T. thermophila* display a plastic capacity sufficient to exceed the range of values observed in the ‘Start’ patch. Seeking further evidence towards the existence of plastic dispersal syndromes represents crucial information: through their context‐dependence, these have the potential to greatly affect the dynamics of metapopulations and their response to environmental changes. Plastic syndromes may increase the occurrence of successful dispersal and tamper the inherent costs of long‐distance movements induced by fragmentation, thus favoring metapopulation persistence and the maintenance of functional connectivity across patchy habitats (Cote et al. 2017; Jacob et al. 2020), a designation that aptly describes the ephemeral feeding spots occupied by *T. thermophila* within freshwater bodies (Doerder and Brunk 2012).

### 4.4 Current Limitations and Futures Uses for the 2D Movement Tracking System

The present study conducted with APIANE used a ‘snapshot’ approach for data collection, shooting very short (10 s) videos at numerous time points throughout 6.5‐h dispersal assays. Though it provides a good population overview, this method limits the detection of individual movement decisions across the landscape; for instance, behavioral decisions occurring over several tens of seconds, such as U‐turns inside the corridor, cannot be detected using short videos. Furthermore, capturing uninterrupted dispersal trajectories from one patch to another remains crucial to go beyond the present data in disentangling the hypotheses of spatial segregation and plasticity at the individual level. Assuming straight swimming at a constant speed of 155 μm/s (averaged data from the five strains), cells would require a minimum of 130 s to traverse the ‘Short’ corridor. To this computed minimal duration, one must add probable delays incurred by dispersal decisions such as conspecific attraction and return tendency, as well as speed and linearity adjustments across time and space, all of which were absent from simulations and likely contributed to large discrepancies between observed and predicted dispersal durations. Therefore, future studies using these tools should aim for continuous footage reaching several (tens of) minutes in length.

A second limitation of our methods lies in the inevitable simplification aspect of artificial microcosms, taken a step further with a strong constraint over the vertical dimension (i.e., the Z‐axis) compared with the three‐dimensional, tube‐based microcosms popularized by Fjerdingstad et al. (2007). In the latter microcosms, the corridor's entrance is additionally characterized by its depth within the tube (usually affixed near the top, largely above the conical base where cellular debris and waste accumulate; Junker et al. 2021). Different strains may prefer to occupy distinct depths inside these tubes, either gathering near or choosing to avoid this particular zone with dense solid matter content; this should affect their probability to encounter the corridor during routine movements and could explain why certain strains behaved quite differently than expected in 2D microcosms (Pennekamp et al. 2014; Junker et al. 2021). In nature, the notion of a dispersal corridor is likely traded for that of a wider ‘matrix’ (sections of the water column that are comparably less hospitable than ‘patches’ offering shelter and an abundance of resource); according to field sampling efforts, such spots should be numerous at greater depths, since decaying vegetation and abundant bacteria would accumulate near the sediment (Doerder and Brunk 2012; Cleven 2004). Dispersal events may thus mostly consist of long distance movements along the length and width of freshwater bodies, rarely occurring beyond those lower zones, consequently minimizing the involvement of the Z‐axis in the natural dispersal dynamics of *T. thermophila* .

The current design of the 2D plates remains relatively simple in both its layout and ease of manufacture. With only minimal adjustments, these novel devices could allow studying the mechanisms behind more complex dispersal patterns reported by previous studies on *Tetrahymena*. For instance, the implementation of branching paths along with localized heating would permit the tracking of cell movements and decisions responsible for habitat choice, which can be based on genotypically determined thermal preference (Jacob et al. 2018; Jacob et al. 2020). Differential cell staining using products that do not interfere with movement or survival may also allow for a plethora of experiments involving genotype mixing where key questions imply strains should be discriminated, e.g., studying cell interactions to understand the impact of kin cooperation on dispersal (Schtickzelle et al. 2009; Jacob et al. 2016), or how information brought by immigrants modifies dispersal decisions (Cote and Clobert 2007; Chaine et al. 2013; Jacob et al. 2015). Dozens of previously characterized laboratory strains of *T. thermophila* are also available (Pennekamp et al. 2014); those may be used to investigate the existence of further dispersal strategies displayed at emigration and immigration, beyond the ‘slope’ and ‘hump’ patterns highlighted in the present study. Finally, other ciliate species on which dispersal studies are frequently performed in microcosms (e.g., *Paramecium caudatum* , *Tetrahymena pyriformis* ) should also benefit from constraining movements within limited depth, which will allow for invaluable behavioral data to be collected (Fellous et al. 2011; Laszlo 2012; Kovács et al. 1994).

## Author Contributions

**Florent Manzi:** conceptualization (equal), data curation (equal), formal analysis (equal), investigation (lead), methodology (equal), project administration (equal), validation (equal), visualization (lead), writing – original draft (lead), writing – review and editing (lead). **Victor Brans:** conceptualization (equal), data curation (equal), formal analysis (equal), methodology (equal), validation (equal), visualization (supporting), writing – review and editing (supporting). **Michaëlla Dacek:** formal analysis (supporting), validation (equal), visualization (supporting), writing – original draft (supporting), writing – review and editing (supporting). **Staffan Jacob:** conceptualization (equal), funding acquisition (supporting), supervision (supporting), visualization (supporting), writing – review and editing (supporting). **Nicolas Schtickzelle:** conceptualization (equal), funding acquisition (lead), methodology (equal), project administration (equal), resources (lead), software (lead), supervision (lead), visualization (supporting), writing – review and editing (supporting).

## Funding

This study was supported by the Fonds de la Recherche Scientifique—FNRS (Belgium) (PDR grant T.0211.19), UCLouvain (Action de Recherche Concertée grant DIVERCE 18–23/095), the Agence Nationale de la Recherche (ANR‐19‐CE02‐0016) and TULIP (Laboratory of Excellence Grant ANR‐10 LABX‐41). This is publication number BRC 439 of the Biodiversity Research Centre at UCLouvain.

## Conflicts of Interest

The authors declare no conflicts of interest.

## Supporting information


**Appendix S1:** Information on *Tetrahymena thermophila* strains.
**Appendix S2:** Preliminary steps to Phase 2 (‘Particle analysis’).
**Appendix S3:** Parameters and quality criteria used for image analysis.
**Appendix S4:** Correction of trajectory linearity values for shortened duration bias.
**Appendix S5:** Parameterization of the null simulation model.
**Appendix S6:** Additional figures and tables for results.

## Data Availability

The data and scripts supporting this study are available from Zenodo: https://doi.org/10.5281/zenodo.15648211.
