# Supplementary material for: Tracking Cell Movement in Two‐Dimensional, Fragmented Microcosms Reveals Dispersal Syndromes and Strategies in Tetrahymena thermophila
Source: Ecol Evol. 2026 Feb 12;16(2):e73092. doi: 10.1002/ece3.73092 (PMC12901667; doi:10.1002/ece3.73092)
Supplement: Supplementary file 1 — Appendix S1: Information on Tetrahymena thermophila strains. Appendix S2: Preliminary steps to Phase 2 (‘Particle analysis’). Appendix S3: Parameters and quality criteria used for image analysis. Appendix S4: Correction of trajectory linearity values for shortened duration bias. Appendix S5: Parameterization of the null simulation model. Appendix S6: Additional figures and tables for results. [file ECE3-16-e73092-s001.docx]

**Appendices S1 – S6**

**Appendix S1: Information on *Tetrahymena thermophila* strains**

**Table S1.** Detailed characterization of the five strains of *Tetrahymena thermophila* used in the experiment. Information about sample isolation was taken from Pennekamp et al., 2014 and the Tetrahymena Stock Centre (<https://sites.wustl.edu/tetrahymena/>). Movement characteristics were retrieved from Pennekamp, 2014. Mean activity rate designates the proportion of cells that changed location over three consecutive frames shot at 1 s intervals.

| **Strain** | **D2** | **D4** | **D11** | **D14** | **D21** |
| --- | --- | --- | --- | --- | --- |
| **Reference** | TSC SD01547 | TSC SD01549 | TSC SD01558 | TSC SD01554 | TSC SD01565 |
| **Isolator** | Doerder | Doerder | Doerder | Doerder | Doerder |
| **Isolation date** | 08/2002 | 06/2003 | 07/2009 | 08/2008 | 07/2009 |
| **Geographic location** | CRWP, PA | SG29, PA | Island Pond #1, NH | SG69-6, PA | Perkins Pond, NH |
| **Mean speed**  **(µm s^-1^)** | 106.84 | 141.88 | 151.69 | 254.98 | 184.21 |
| **Mean activity**  **rate** | 0.63 | 0.69 | 0.66 | 0.69 | 0.71 |

**Appendix S2: Preliminary steps to Phase 2 (‘Particle analysis’)**


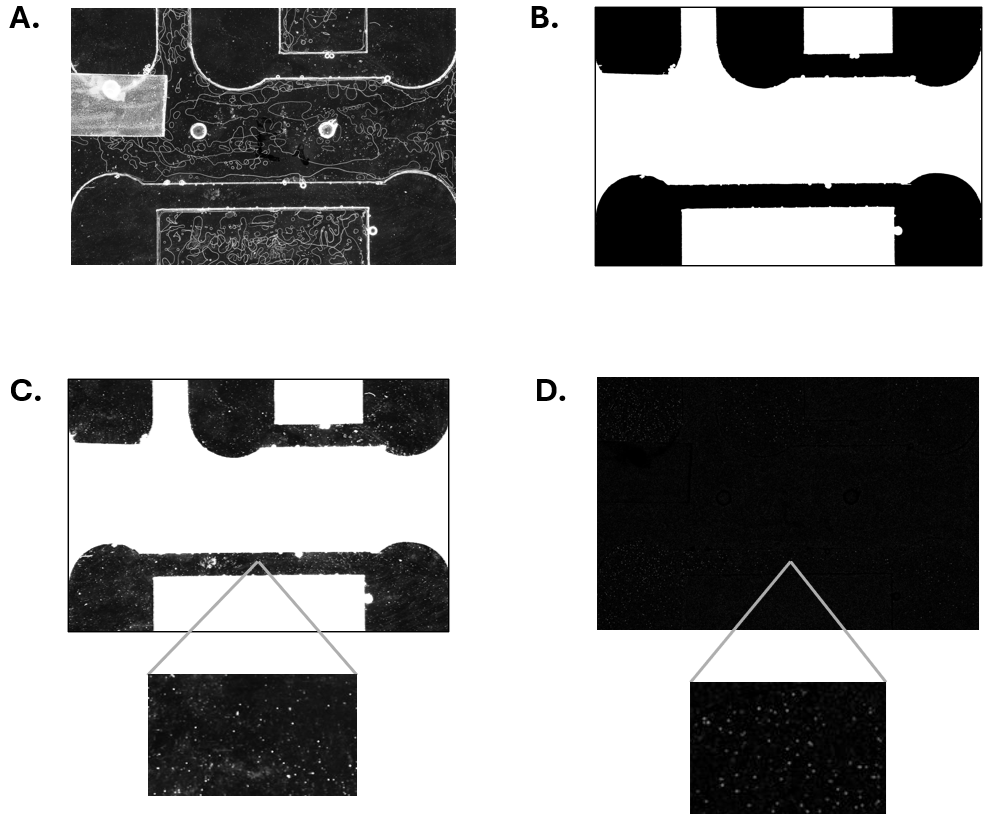


**Figure S1.** Image processing steps necessary to construct a series’ background, which facilitates particle identification and tracking. **A.** The ‘average picture’ was created using the mean value of grey level for each pixel across all 75 frames in a 10-second picture series. Visible as a bright rectangle on the left side is part of the polyethylene membrane covering the ‘Control’ patch’s exit point for liquid and air. **B.** A ‘mask’ was generated from the middle (38^th^) picture in the series. Parts of the picture that did not belong to the carved out landscapes were selected, manually adjusted if needed, then deleted using Fiji (here shown in white). **C.** The series’ background resulted from a combination of the average grey level picture (A) and the mask (B). This allowed the deletion of any part of the image that was constant across all pictures, leaving only moving elements, consisting in their vast majority of *T. thermophila* cells. The enlarged section shows grey and white elements that are part of the background but are not identified as *T. thermophila*. **D.** Parts of the plate hidden by the mask become black during image processing, and the background (C) is subtracted from each picture in the series. The enlarged section shows that only *T. thermophila* cells remain as whiter areas before particle identification and trajectory analysis, which greatly reduces processing time and improves results.

**Appendix S3: Parameters and quality criteria used for image analysis**

**Table S2.** List of quality criteria used during the ‘Particle analysis’ and ‘Tracking’ steps of image processing.

| **Parameter** | **Value** | **Definition** |
| --- | --- | --- |
| **Step 2 (‘Particle analysis’) – parameters for particle identification** | | |
| Grey scale threshold | 40 | Value to split pixel grey scale into ‘black’ vs. ‘white’ |
| Minimum size | 40 | Minimum size (in white pixels) to consider a particle |
| Maximum size | 500 | Maximum size (in white pixels) to consider a particle |
| **Step 2 (‘Particle analysis’) – postprocessing quality criteria:**  **each sample (burst of 75 frames) is manually checked if any of these criteria is met** | | |
| Abnormal particle abundance | 1.20 | A frame is considered an ‘outlier’ in the series if its particle abundance differs from the average by more than 20% |
| Abnormal grey value | 1.10 | A frame is considered an ‘outlier’ in the series if its average grey level value differs from the average by more than 10% |
| **Step 3 (‘Tracking’) – parameters for trajectory reconstruction** | | |
| Link range | 8 | Range of consecutive frames that are taken into account to find candidate particles for trajectory reconstruction |
| Displacement | 25 | Maximum displacement (in pixels) between two frames for a given particle to be considered a candidate |
| **Step 3 (‘Tracking’) – postprocessing quality criteria: a trajectory is discarded if any of these criteria is not met** | | |
| Gross displacement | 100 | Minimum gross displacement (in μm) from the cell’s starting point |
| Duration | 1 | Minimum duration of a trajectory (in seconds) |
| Detection frequency | 0.6 | Minimum fraction of the frames on which the particle is detected |
| Median step length | 3 | Minimum median of the step length (in μm) between two consecutive frames |

**Appendix S4: Correction of trajectory linearity values for shortened duration bias**

During the step of particle tracking, many movement trajectories are reconstructed from fewer than
10 s (75 frames). There are two main technical reasons for that. First, the tracking algorithm starts a new trajectory for a cell when it cannot discriminate among several candidate cells on the next frame. This occurs particularly when a cell collides with another cell or with an artifact while other cells are located nearby. Second, individuals may enter or leave the camera field of view during shooting. The linearity (net displacement / gross displacement ratio) of such shortened trajectories is expected to be biased upwards: the shorter the trajectory, the more linear it will appear, the limit being a trajectory made of two positions being always a straight line. This was indeed observed in our data, with a clear linear relation (Figure S2A). Thus, to allow for proper comparison of linearity across trajectories of varying duration, a detrending correction method was devised to compute the expected linearity for each observed trajectory as if it had been measured over the full 10 s.

To parameterize the correction function, we used a dataset of 879556 trajectories recorded in the ‘Control’ patch of 2D plates within the present experiment and others using strictly identical experimental conditions. Linearity was first logit-transformed to be expressed in [-∞ ; +∞] rather than in [0;1] and the correction function was estimated by fitting a simple linear regression. To ease interpretation and later correction, linearity (and correction function) was expressed in a new referential (Figure S2B) by moving the origin (0,0) position:

- along the x-axis (duration), subtracting the maximal trajectory duration value (i.e., 75 frames) places the 0 at the theoretically recorded duration, with x being the number of missing frames;
- along the y-axis (logit-transformed linearity), subtracting the predicted linearity value when trajectory duration is maximal (i.e., intercept of the linear regression: -0.39) means this new y expresses the ‘linearity excess (logit-transformed) when trajectory duration is shorter than full length’ (i.e., 75 frames, corresponding to the 0 of this new y axis).

The estimated correction function was then used to correct the observed linearity value for each recorded trajectory in the experiment by:

1. computing the linearity excess predicted according to the number of missing frames:

$$PredictedExcess=0.0403\cdot MissingFrames$$

2. subtracting this excess from the observed (logit-transformed) linearity:

$$CorrectedLogitLinearity=LogitLinearity- PredictedExcess$$

3. expressing linearity in its original [0;1] by back transforming with the inverse logit:

$$CorrectedLinearity=\frac{1}{1+e^{-(CorrectedLogitLinearity))}}$$

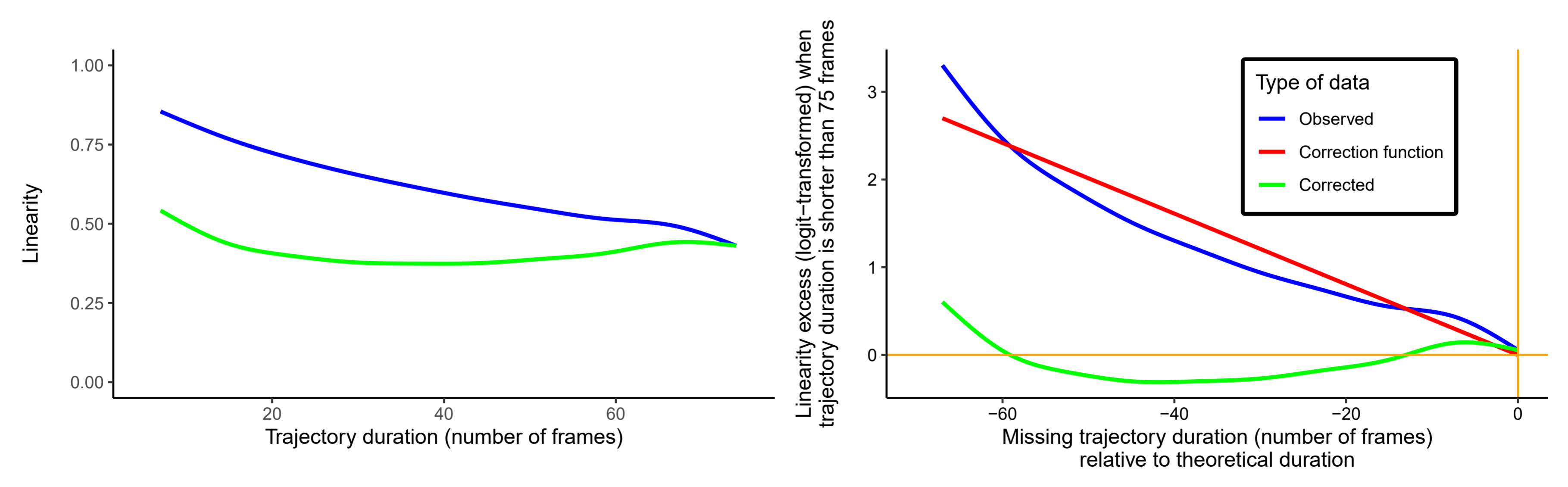


**Figure S2.** **A**. In observed trajectories, measured linearity (in blue) shows a linear trend upwards when their duration is shorter, a trend that disappears once the correction is applied (in green). **B**. The same data is depicted in the modified referential used to estimate the correction function (in red), where [0,0] (in orange) corresponds to a trajectory of full length with no correction needed. Observed data were smoothed using GAM (via ‘geom_smooth’ with default smoothing method) to accommodate for their very high number (879556 trajectories).

**Appendix S5: Parameterization of the null simulation model**

To complement experimental data, a simulation model was built using NetLogo software version 6.3.0 (Wilensky, 1999). The aim of this model was to generate cell trajectories and predict dispersal within the landscape under the assumption that no ‘movement decision’ is taken by cells. Individual agents (or ‘turtles’ in NetLogo vocabulary, i.e., simulated *Tetrahymena* cells) were generated as distinct points capable of travelling across a set of virtual X and Y coordinates, within a landscape shaped identically as real dispersal systems, including the ‘Control’, ‘Short’ and ‘Long’ landscapes (the ‘world’). At the start of each simulation, 1000 turtles were generated within each of those locations, all spawning at the precise coordinate corresponding to real systems’ entry points. Movement behavior of turtles was parameterized using real data from the ‘Control’ patch: each turtle was assigned to a random file containing the movement speed and turning angle values corresponding to each frame from the trajectories of an experimental cell (trajectories below 4 seconds were filtered out). Turtles were programmed to execute 10 pairs of ‘speed’ and ‘turning angle’ values from a given row in the reference file, before switching to another random set of 10 consecutive pairs within the same file. Within the simulation, one ‘tick’ (unit of time) represents 0.133 seconds, as real videos were taken at 7.5 frame-per-second. At each tick after the start of a simulation, turtles were asked to change positions according to the pair of movement values randomly selected. Both movement parameters were adapted for each simulated *Strain,* depending on experimental data observed in the ‘Control’ patches across all time points of the experiment.

Turtles were programmed to ‘bounce’ when colliding with the border of the virtual landscape but ignored collisions with other cells. Bouncing means that cells are reflected from the wall at the same angle at which they approached it; cells that are heading perpendicularly to a wall will randomly turn to the left or to the right, with an angle between 0 and 90 degrees relative to the wall. In such an instance, this bouncing behaviour replaces the normally selected turning angle value, however speed is maintained as previously mentioned. Overall, dispersal movements in the simulations were only driven by basic movement parameters and the geometry of the landscape. Contrary to experimental data, simulations could not involve any form of individual dispersal ‘decision’ based on either internal state variables, environmental cues, or interactions with conspecifics. Simulations (i.e., ‘dispersal assays’) ran continuously from t = 0 to t = 396 min, corresponding to the time at which experimental assays were terminated. Six replicate simulations were run per strain. To properly compare abundance and dispersal data, trajectory data were only recorded within the equivalent of the camera field of view within the virtual landscapes (Figure S3). Snapshots of data were recorded as raw data files every 12 min, containing sets of trajectories lasting up to 75 frames (10 s).


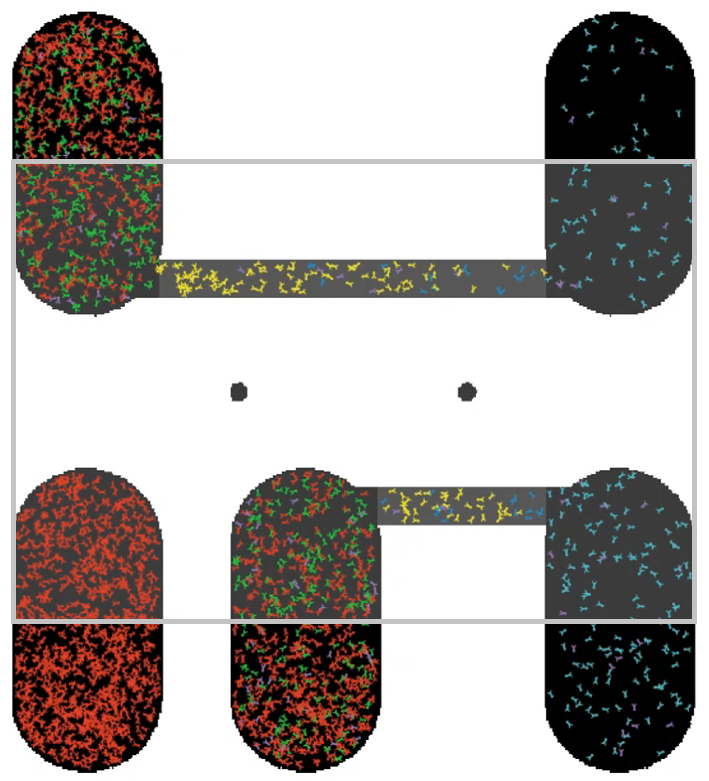


**Figure S3.** Screenshot from a simulated run of the ‘null’ model built using NetLogo software. The ‘Control’, ‘Short’ and ‘Long’ landscapes are reproduced with identical proportions to the real experimental microcosms. The light grey frame depicts the area within which data are recorded and corresponds to the camera field of view in real experimental setup. Turtles (i.e., simulated *Tetrahymena* cells) are coloured depending on their status throughout the simulated dispersal assay. Red: cells that have never left the ‘Control’ or ‘Start’ patch. Yellow: cells that have entered the corridor. Green: cells that have returned to the ‘Start’ patch after having reached the corridor. Light blue: cells that have entered the ‘Target’ patch. Dark blue: cells that have returned to the corridor after having reached the ‘Target’ patch. Purple: cells that have returned all the way to the ‘Start’ patch after having reached the ‘Target’ patch.

**Appendix S6: Additional figures and tables for Results**


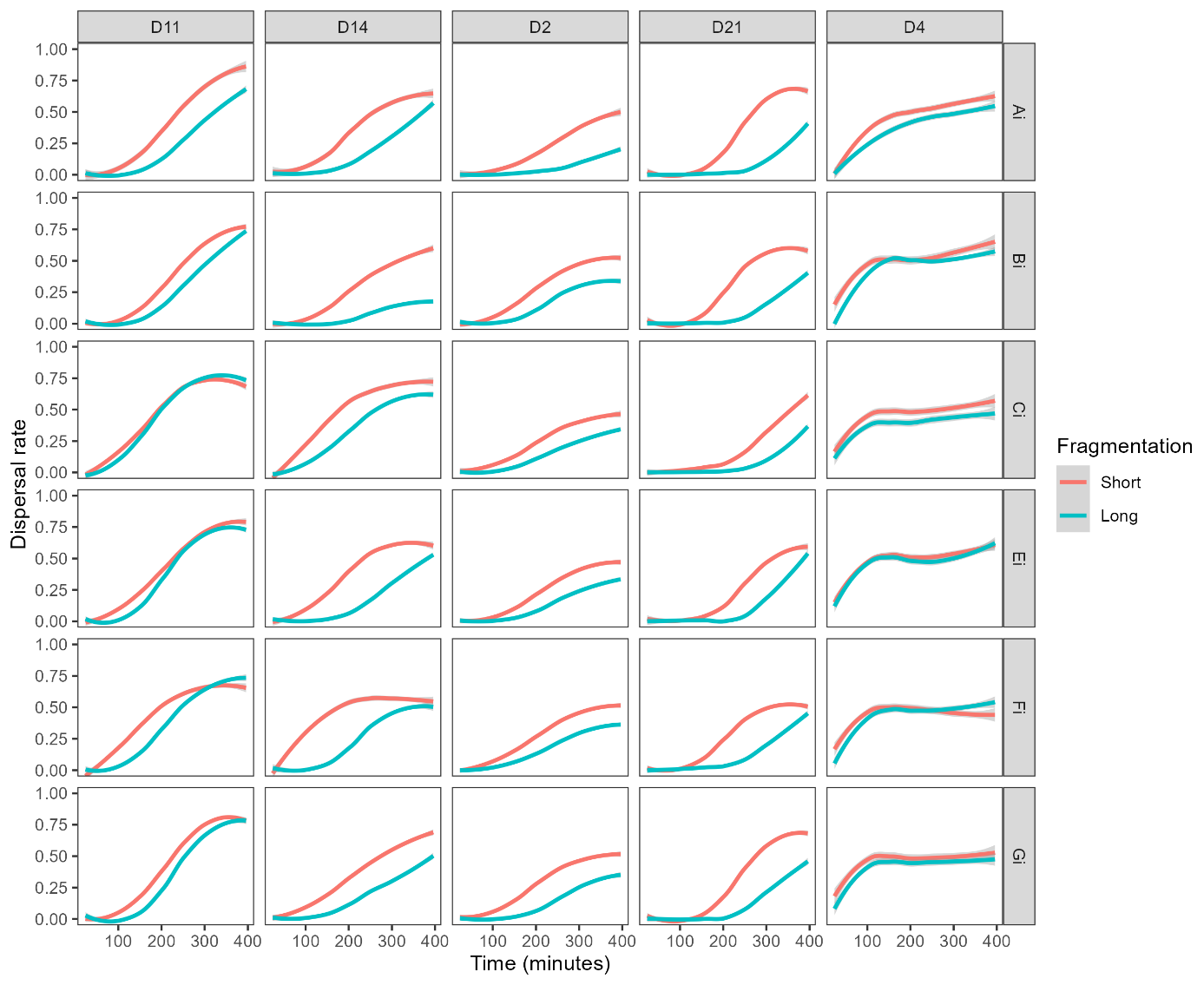


**Figure S4.** Dispersal rate is depicted over time, at two levels of landscape *Fragmentation*, for all 60 populations assayed during the experiment. Thirty-two time points were recorded over 396-minute long dispersal assays, data smoothing was applied with the LOESS method. Sets of ‘Short’ and ‘Long’ landscapes that belong to the same 2D plate are depicted on the same panel.

**Table S3.** Two-way ANOVA table describing the effects of *Fragmentation* (fixed effect), *Strain* identity (random effect) and their interaction (random) on the dispersal-related traits: A) dispersal rate at termination and B) time until half dispersal. Significant *P-value*s (≤ 0.05) are highlighted in bold.

| A) Dispersal rate at termination | | | | | | |
| --- | --- | --- | --- | --- | --- | --- |
|  | Degrees of freedom | | Sum of squares | Mean square | *F*-value | *P-*value |
| Fragmentation | 1 | | 0.26 | 0.26 | 10.38 | **0.032 *** |
| Strain | 4 | | 0.74 | 0.18 | 35.42 | **<0.0001 ***** |
| Fragmentation × Strain | 4 | | 0.100 | 0.025 | 4.82 | **0.002 **** |
| Residuals | 50 | | 0.26 | 0.005 |  |  |
| B) Time until half dispersal | | | | | | |
| Fragmentation | 1 | 27993.6 | | 27993.6 | 13.62 | **0.021 *** |
| Strain | 4 | 320366.4 | | 80091.6 | 91.58 | **<0.0001 ***** |
| Fragmentation × Strain | 4 | 8222.4 | | 2055.6 | 2.35 | 0.067 |
| Residuals | 50 | 43728.0 | | 874.6 |  |  |

**Table S4.** Three-way ANOVA table describing the effects of *Zone* (fixed effect), *Fragmentation* (fixed effect), *Strain* identity (random effect), and their interactions on the following traits: A) swimming speed, B) linearity, C) cell size, and D) cell shape. Significant *P-value*s (≤ 0.05) are highlighted in bold.

| A) Swimming speed | | | | | | |
| --- | --- | --- | --- | --- | --- | --- |
|  | Degrees of freedom | | Sum of squares | Mean square | *F*-value | *P-*value |
|  |  | |  |  |  |  |
| Zone | 2 | | 76380.7 | 38190.3 | 15.136 | **0.002 **** |
| Fragmentation | 1 | | 894.2 | 894.2 | 2.328 | 0.202 |
| Zone × Fragmentation | 2 | | 3148.5 | 1574.3 | 10.851 | **0.005 **** |
| Strain | 4 | | 225408.9 | 56352.2 | 534.56 | **<0.0001 ***** |
| Zone × Strain | 8 | | 20184.6 | 2523.1 | 23.934 | **<0.0001 ***** |
| Fragmentation × Strain | 4 | | 1536.2 | 384.0 | 3.643 | **0.007 **** |
| Zone × Fragmentation × Strain | 8 | | 1160.7 | 145.1 | 1.376 | 0.211 |
| B) Trajectory linearity | | | | | | |
|  |  |  | |  |  |  |
| Zone | 2 | 0.0612 | | 0.0306 | 1.696 | 0.243 |
| Fragmentation | 1 | 0.0037 | | 0.0037 | 5.640 | 0.076 |
| Zone × Fragmentation | 2 | 0.0109 | | 0.005 | 9.525 | **0.008 **** |
| Strain | 4 | 1.0887 | | 0.272 | 549.591 | **<0.0001 ***** |
| Zone × Strain | 8 | 0.1443 | | 0.018 | 36.445 | **<0.0001 ***** |
| Fragmentation × Strain | 4 | 0.0026 | | 0.0007 | 1.332 | 0.261 |
| Zone × Fragmentation × Strain | 8 | 0.0046 | | 0.0006 | 1.152 | 0.332 |
| C) Cell size | | | | | | |
| Zone | 2 | 249427.5 | | 124713.7 | 14.409 | **0.002 **** |
| Fragmentation | 1 | 134221.8 | | 134221.8 | 6.254 | 0.067 |
| Zone × Fragmentation | 2 | 5012.7 | | 2506.3 | 0.930 | 0.433 |
| Strain | 4 | 2650942.2 | | 662735.5 | 275.893 | **<0.0001 ***** |
| Zone × Strain | 8 | 69244.5 | | 8655.6 | 3.603 | **<0.001 ***** |
| Fragmentation × Strain | 4 | 85852.8 | | 21463.2 | 8.935 | **<0.0001 ***** |
| Zone × Fragmentation × Strain | 8 | 21555.3 | | 2694.4 | 1.122 | 0.352 |
| D) Cell shape | | | | | | |
| Zone | 2 | 0.073 | | 0.036 | 7.347 | **0.015 *** |
| Fragmentation | 1 | 0.007 | | 0.007 | 6.642 | 0.062 |
| Zone × Fragmentation | 2 | 0.0001 | | 0.00005 | 0.214 | 0.812 |
| Strain | 4 | 1.363 | | 0.341 | 513.677 | **<0.0001 ***** |
| Zone × Strain | 8 | 0.040 | | 0.005 | 7.443 | **<0.0001 ***** |
| Fragmentation × Strain | 4 | 0.004 | | 0.001 | 1.578 | 0.183 |
| Zone × Fragmentation × Strain | 8 | 0.002 | | 0.0002 | 0.368 | 0.936 |


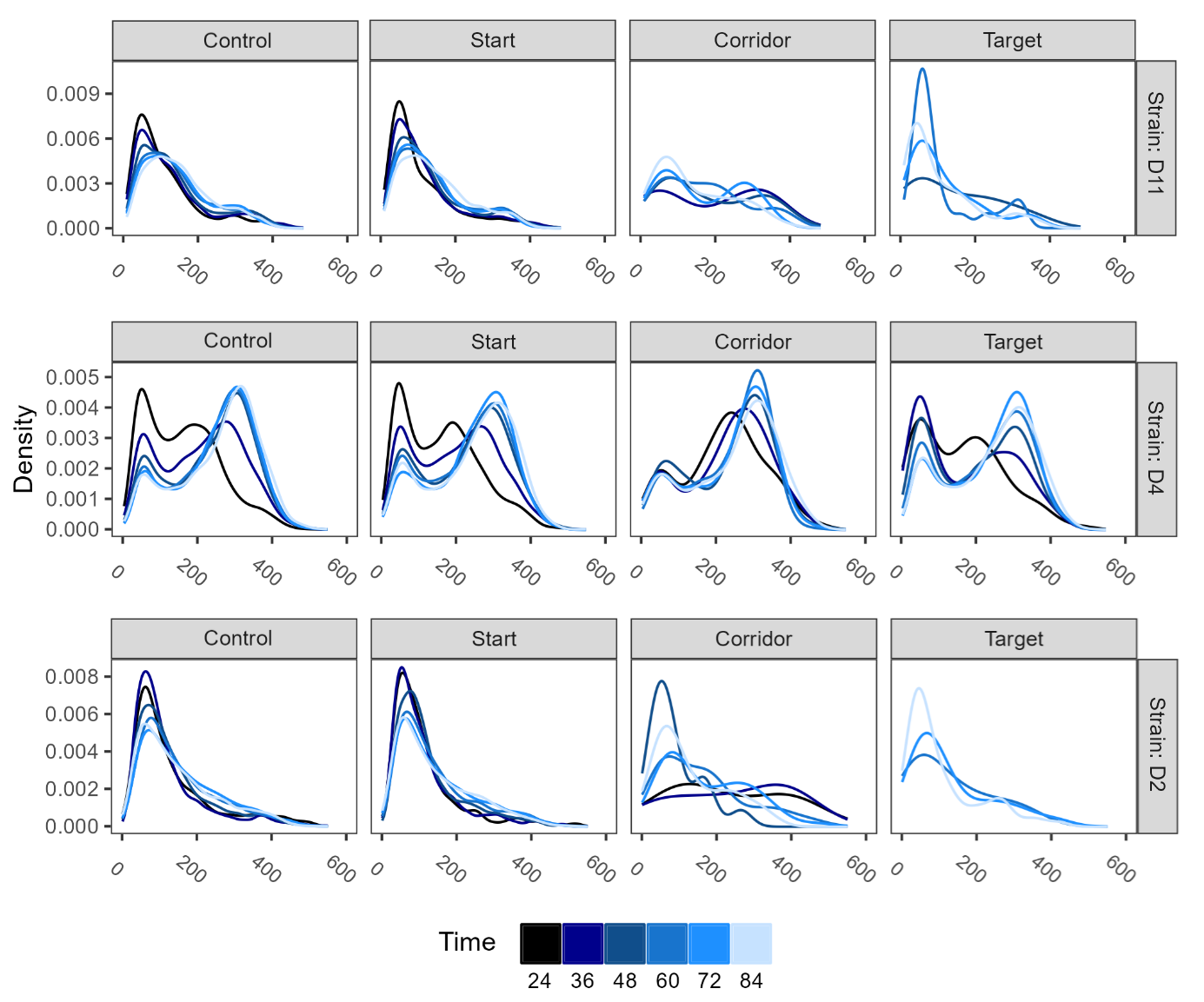


**Figure S5.** Swimming speed distribution was visualized with a color gradient corresponding to the first six time points of the experiment. Data for strains D11, D4, and D2 within the ‘Control’ patch and zones of the ‘Short’ landscape are depicted. Distribution in the ‘Control’ and ‘Start’ patches were nearly identical for a given strain: starting with a peak at low velocity at the start of shooting (t = 24 min), data was later skewed towards higher swimming speed values, with a varying magnitude across strains (an extreme example being displayed by strain D4). This indicates that similar shifts in the distribution of phenotypic traits can emerge over time in both locations, even in the absence of cell departure.


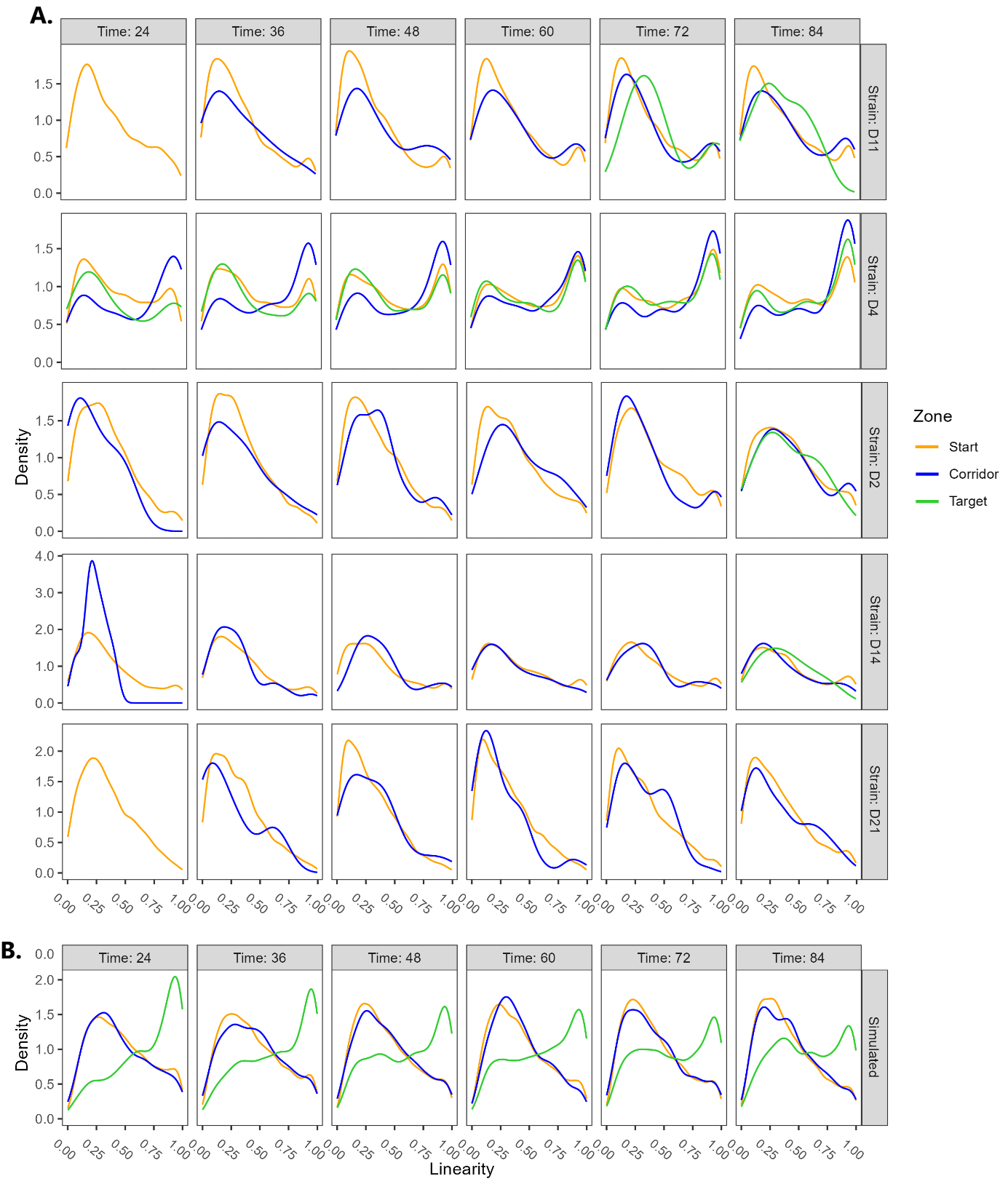
**Figure S6. A.** Observed distribution of trajectory linearity over the first six time points of the experiment. Only data from the ‘Short’ fragmentation level are depicted. **B.** Simulated distributions were generated by the null model (data from all five ‘simulated’ strains were pooled).
